# Evolution of CPEB4 Dynamics Across its Liquid–Liquid Phase Separation Transition

**DOI:** 10.1021/acs.jpcb.1c06696

**Published:** 2021-11-17

**Authors:** Manas Seal, Chandrima Jash, Reeba Susan Jacob, Akiva Feintuch, Yair Shalom Harel, Shira Albeck, Tamar Unger, Daniella Goldfarb

**Affiliations:** ^†^Department of Chemical and Biological Physics, ^‡^Department of Biological Regulation, ^§^Department of Structural Biology, and ^∥^Department of Life Sciences Core Facilities, Weizmann Institute of Science, 7610001 Rehovot, Israel

## Abstract

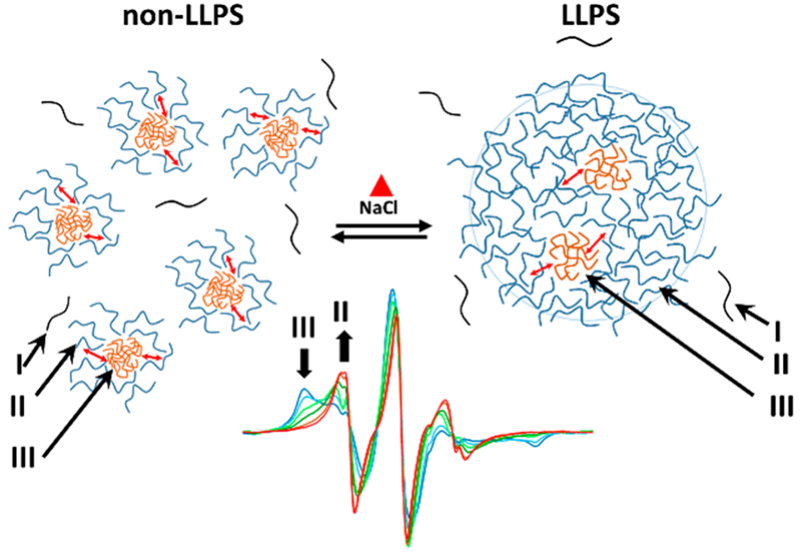

Knowledge about the structural and dynamic properties of proteins that form membrane-less organelles in cells via liquid–liquid phase separation (LLPS) is required for understanding the process at a molecular level. We used spin labeling and electron paramagnetic resonance (EPR) spectroscopy to investigate the dynamic properties (rotational diffusion) of the low complexity N-terminal domain of cytoplasmic polyadenylation element binding-4 protein (CPEB4_NTD_) across its LLPS transition, which takes place with increasing temperature. We report the coexistence of three spin labeled CPEB4_NTD_ (CPEB4*) populations with distinct dynamic properties representing different conformational spaces, both before and within the LLPS state. Monomeric CPEB4* exhibiting fast motion defines population **I** and shows low abundance prior to and following LLPS. Populations **II** and **III** are part of CPEB4* assemblies where **II** corresponds to loose conformations with intermediate range motions and population **III** represents compact conformations with strongly attenuated motions. As the temperature increased the population of component **II** increased reversibly at the expense of component **III**, indicating the existence of an **III** ⇌ **II** equilibrium. We correlated the macroscopic LLPS properties with the **III** ⇌ **II** exchange process upon varying temperature and CPEB4* and salt concentrations. We hypothesized that weak transient intermolecular interactions facilitated by component **II** lead to LLPS, with the small assemblies integrated within the droplets. The LLPS transition, however, was not associated with a clear discontinuity in the correlation times and populations of the three components. Importantly, CPEB4_NTD_ exhibits LLPS properties where droplet formation occurs from a preformed microscopic assembly rather than the monomeric protein molecules.

## Introduction

In the past decade, it has been shown that many cellular membrane-less organelles form via liquid–liquid phase separation (LLPS),^1−6^ where defined sets of proteins and nucleic acids coexist in dilute (bulk) and condensed (droplet) phases in cells.^4,7^ Many proteins undergoing LLPS are intrinsically disordered (IDPs) or contain extended low complexity domains (LCDs),^8^ which are also implicated in disorders like amyotrophic lateral sclerosis (ALS), frontotemporal dementia (FTD), and Alzheimer’s disease (AD).^7^ LLPS driving forces rely on multivalent intra- and intermolecular contacts, including charge–charge, cation−π, π–π, or hydrophobic interactions between different protein residues or protein–nucleic acid contact sites.^9−11^ LLPS can be regulated by a modulation of these interactions; for example phosphorylation or dephosphorylation, which changes the charge distribution in the protein, has been shown to eliminate LLPS all together, or in turn to promote it.^12,13^ Important aspects of LLPS, which are not yet fully understood, are related to the molecular level behavior of the protein in the condensed phase as compared to the dilute phase and its relation to the underlying mechanism of LLPS. Solution-state nuclear magnetic resonance (NMR) techniques are very useful to study protein structure, inter- and intramolecular interactions, local chain dynamics, and translational diffusion and have therefore been used to investigate proteins in condensed and dilute phase under LLPS conditions.^12,14,15^ However, the study of the condensed phase becomes challenging due to line broadening as a consequence of slow motion and other limitations imposed by the low sensitivity.^14^ In this work, we applied EPR (electron paramagnetic resonance) spectroscopy combined with site specific nitroxide spin labeling to resolve the dynamic properties of the N-terminal domain of human cytoplasmic polyadenylation element (CPE) binding-4 protein (CPEB4_NTD_) in its LLPS and non-LLPS states and across the phase transition. Specifically, we aimed at identifying a structural/dynamic switch that can be associated with the LLPS transition. The nitroxide continuous wave (CW) EPR spectrum is particularly sensitive to its environment, and it gives information on backbone and side chain fluctuation at the labeling site at a time scale that can complement NMR measurements.^16^ It is insensitive toward protein size, and use of a lower sample volume and concentration gives important advantages. The EPR line shape reports site specifically on the degree of averaging of anisotropic magnetic interactions via rotational diffusion with correlation times (τ_c_) in the range of 10^–6^–10^–10^ seconds and can resolve different coexisting populations with different τ_c_ values.^16^ Accordingly, EPR spectroscopy has been extensively used to study the dynamics associated with membranes,^17,18^ liquid crystals,^19^ micellar solutions,^20^ proteins^16,21^ and LLPS in polymers^16,22^ and proteins.^23−26^ Fluorescence techniques are also very useful for studying dynamic and structural properties of proteins and provide information complementary to that obtained by EPR and NMR in terms of the time scales observed and the degree of structural resolution. In the context of LLPS FRAP (fluorescence beaching after photobleaching) has been very useful in determining the diffusion time in and out of the droplets.^27^ Fluorescence correlation spectroscopy (FCS) provides translational diffusion times, as opposed to the rotational correlation times provided by EPR. Single molecule FRET (Förster resonance energy transfer) spectroscopy probes conformational fluctuation in the range of 2–8 nm and protein motions in the range of 1 ns to 1 s at the level of individual molecules.^28^ The EPR analog of FRET is the double-electron electron resonance (DEER) experiment,^29^ which provides distance distributions between spin labels in frozen solution. In this work, we used DEER to probe intermolecular spin–spin interactions.

CPEB4 is a member of the CPEB family of proteins,^30,31^ which are involved in the translational regulation of poly-A tails in mRNAs.^31,32^ The protein harbors a disordered N-terminal domain (NTD)^33^ followed by two conserved RNA recognition motifs (RRMs) and a C-terminal zinc binding domain.^34,35^ The NTD has been associated with impaired neuronal development and defects in motor axons in mice.^33^ It has recently been shown that the activity of *Xenopus* CPEB4 is regulated by reversible phosphorylation at multiple NTD sites.^36^ Whereas phosphorylated CPEB4 is functionally active and mediates cytoplasmic polyadenylation, the nonphosphorylated protein undergoes LLPS *in vivo* and is inactive.^33,36^ Moreover, it has been reported that the NTD domain of *Xenopus* CPEB4 undergoes LLPS also *in vitro*, but the mechanism of LLPS has not been studied at the molecular level.^36^ Unlike other proteins undergoing LLPS,^10,37^ the amino acid sequence of human CPEB4_NTD_ is nondegenerate and contains alternating segments of positively and negatively charged residues along with a significant number of hydrophobic and aromatic residues (see Supporting Information, Figure S1). We labeled CPEB4_NTD_ with a nitroxide spin label at the naturally occurring cysteine residue at position 441, referred to as CPEB4*, (see Figure S1, Supporting Information for details) and studied the dynamic properties of recombinant, nonphosphorylated CPEB4* across multiple LLPS and non-LLPS conditions using EPR spectroscopy. These were complemented and correlated with macroscopic characterization of LLPS by optical microscopy and absorption.

## Experimental and Methods

### Sample Preparation

#### CPEB4_NTD_ Expression and Purification

The human CPEB4_NTD_ plasmid (isoform 2)^38^ was received from Professor. Xavier Salvatella, IRB, Barcelona. The plasmid was designed in a pET-30a vector to express the N-terminal domain of CPEB4 (1–448) without a His-tag and cleavage site. The protein was expressed in BL21(DE3) *Escherichia coli* cells for 3 h at 37 °C, and the bacteria were lysed using sonication in lysis buffer (see Table S1 for all the buffer details). The protein was found in inclusion bodies (IB), which were washed with IB wash-I and -II buffers and finally solubilized in a IB-resolubilization buffer. The protein was purified using a Ni^2+^–NTA column, 2–3 column volume washes with Ni-buffer-A and finally followed by elution with Ni-buffer-B. The protein was further purified using a Superdex-200 size exclusion column using size exclusion buffer. The purified protein (checked by SDS-PAGE) was flash frozen and stored at −80 °C. The C441S/G320C CPEB4_NTD_ mutant was purified using the same protocol. The yield was 5–7 mg from 2 L of culture.

#### Labeling of CPEB4_NTD_

For EPR spectroscopy, we labeled CPEB4_NTD_ with (1-oxyl-2,2,5,5-tetramethyl-Δ3-pyrroline-3-methyl) methanethiosulfonate (MTSL, Toronto Research Chemicals). CPEB4_NTD_ has only one intrinsic cysteine residue at position 441. This residue was labeled with MTSL, and we referred to it as CPEB4*. CPEB4_NTD_ was concentrated to above 100 μM, and the size exclusion buffer was replaced with 25 mM Tris HCl pH 7.2, 3 M GdmCl using PD Spin trap column (GE Healthcare). Here 20 equiv of MTSL (using a 50 mM stock solution in DMSO) was immediately added to the protein and was allowed to react at room temperature for 2–3 h. Excess spin label was removed using a 5 mL HiTrap desalting column in FPLC. The elution buffer used for obtaining a denatured protein was 25 mM TrisHCl pH8 and 3 M GdmCl, whereas for the native state the elution buffer was 25 mM TrisHCl pH8 and 100 mM NaCl (Table S1). The concentration of the protein was determined by UV–vis and the spin concentration from EPR. The labeling efficiency was 95 ± 5%.

For the phosphorylation experiments, CPEB4_NTD_ was labeled with 3-maleimidoproxyl (MSL) following the same procedure as for MTSL. MSL was preferred over MTSL as it forms a C–S bond, resistant to reduction by DTT, present in the commercial kinases. This construct is referred as CPEB4_NTD_-MSL.

#### Phosphorylation

CDK1/Cyclin A2 (C0244) and ERK1 (SRP5282) (Sigma-Aldrich) stock solutions were in kinase dilution buffer prepared by a 5 times dilution from kinase assay buffer (25 mM MOPS, pH 7.2, 12.5 mM glycerol 2-phosphate, 25 mM MgCl_2_, 5 mM EGTA, 2 mM EDTA, and 0.25 mM DTT) as mentioned by the manufacturer. Final protein, CDK1, ERK1, and ATP concentrations in the reaction mixture were 1 μM, 14.4 nM (2 ng/μL), 13.8 nM (1 ng/μL) and 1 mM, respectively. The mixture was incubated at 37 °C for 30 min, 1 h, and 2 h and then flash-frozen in liquid nitrogen. The mixture was subjected to mass spectrometry analysis (50 μL of 1 μM CPEB4_NTD_-MSL). The mass spectrometry data showed identical results for the three different reaction times. For EPR measurements, the CPEB4-MSL concentration was 20-25 μM, with a volume of 25 μL, and the CDK1 and ERK1 concentrations were 180 and 173 nM, respectively.

#### Separation of Dilute Phase

To separate the dilute phase from the condensed phase,^27^ 20 μL of a 112 μM CPEB4* solution was incubated at room temperature for 15 min and then centrifuged at room temperature at 10000*g* in an Eppendorf tube for 30 min. The condensed phase made a pellet at the bottom. The supernatant was carefully collected into a capillary, and the EPR spectrum was recorded.

#### Measurements with 1,6-Hexanediol (HD)

HD was purchased from ACROS ORGANICS (Fisher Scientific), and a 50% w/v stock solution was prepared in 25 mM TrisHCl pH8 and 100 mM NaCl. The effect of HD on CPEB4* was checked with EPR spectroscopy by varying the percentage from 0.5% to 10% for a CPEB4* concentration of 20 μM by using the stock solution of HD.

#### Sample Preparation for Pulse EPR Measurements

These measurements are carried out at low temperatures and therefore require the addition of a cryo-protectant. Accordingly, samples were prepared in 10% (V/V) glycerol and were flash frozen in an isopentane bath cooled with liquid nitrogen. We confirmed that this did not disrupt the droplet formation nor changed the EPR spectrum (Figure S2A–C). Increasing the amount of glycerol to 20% lead to aggregation. To trap the sample in the phase-separated state the sample was incubated at room temperature for 15–20 min and then flash-frozen. To trap the sample prior to the LLPS transition, it was incubated over ice for 15–20 min and then flash-frozen in cooled isopentane, and the sample preparation was carried out in the cold room (4 °C).

### Methods

#### Microscopy

Droplet formation of CPEB4* was monitored by differential interface contrast (DIC) on a Leica DMI8 microscope with a 63× objective (glycerol immersion) or a 100× objective (oil immersion). Then, 1–2 μL of ice-cold CPEB4* was spotted on an imaging chamber generated by attaching a coverslip to a clean glass slide using a thin double-sided tape. For the low temperature images, the slides were kept over ice for 15–20 min and immediately transferred to the microscope. The mounting of the slide and focusing took about 30–45 s. The slide was allowed to warm at room temperature, and images were collected at different times. Images were processed using Fiji software (NIH).

#### Absorption Spectroscopy

The absorption spectra were recorded on an Agilent 8453 spectrophotometer coupled to a variable temperature cuvette holder. Either 100 μL of protein was placed in a thin cuvette with a 1 mm path length or 50 μL of protein was used with a cuvette having a path length of 10 mm. For every temperature, 15–30 min was used to allow equilibration.

#### Dynamic Light Scattering (DLS)

The polydispersity index of the solution across the LLPS of CPEB4* was obtained by DLS measurements using a Malvern’s Zetasizer Nano ZSP with backscatter detection system at 173° angle. A minimum of three measurements were recorded for each data point. ZEN0040 disposable cuvettes with a capacity of 40 μL of sample were used. The equilibration time at each temperature was about 15–30 min. The polydispersity index was obtained from the output data form the software of each measurement and each point is an average of three to five data sets.

#### Mass Spectrometry

Mass spectrometry for the phosphorylated CPEB4_NTD_-MSL was carried out at the mass spectrometry facility at The De Botton Protein Profiling institute of the Nancy and Stephen Grand Israel National Center for Personalized Medicine, Weizmann Institute of Science. The samples were subjected to tryptic digestion or tryptic + chemotryptic digestion using an S-trap. The resulting peptides were analyzed using nanoflow liquid chromatography (nanoAcquity) coupled to high resolution, high mass accuracy mass spectrometry (Q Exactive HF). The data were analyzed by MS/MS (using Byonic software) and also based on MS intensity.

#### EPR Spectroscopy

Continuous wave (CW) EPR spectra were recorded on an Elexsys E500 X-Band (9.5 GHz) Bruker spectrometer with a high sensitivity resonator. Samples were placed into capillaries of 0.84 mm o.d. and 0.6 mm i.d. filled up a height of 2 cm. Both ends of the capillaries were sealed with crytoseal. For low concentration samples, three capillaries were used. The measurements were performed using a field modulation amplitude of 0.1 mT, a scan range of 15 mT, and a microwave power of 20 mW. Each scan was 42 s, and at least 25 scans were accumulated for high concentrations, whereas for low concentrations (10 μM and below), 100 to 150 scans were collected. For the variable temperature experiments in between 2 and 45 °C a temperature controller from Eurotherm was used with a stability of ±1 °C. The equilibration time was 15–30 min. All the spectra were simulated using the “Chili” routine of Easyspin.^39^ The parameters for simulations are given in Supporting Information, Tables S2 and S3.

DEER and echo decay measurements were carried out at W band (94.9 GHz) on a home-built spectrometer^40−42^ at 25 K and a concentration of 80 μM for the denatured sample 60 μM for LLPS and non-LLPS sample. Two pulse echo decays were recorded with a Hahn echo sequence (π/2−τ–π–τ-echo) using π/2 and π pulses of 17.5–20 ns and 35–40 ns, respectively. DEER measurements were recorded using the four-pulse DEER sequence with a chirp pump pulse.^42,43^ The maxima of nitroxide was set to 94.9 GHz. The observer pulses were set to 94.83 GHz, and the π/2 and π pulse durations were 35 and 70 ns, respectively. The pump pulse was a 100 MHz chirp pulse with the range of 94.88–94.98 GHz starting at an offset of 50 MHz from the observer pulses to prevent overlap effect. The duration of the pump pulse was 128 ns. The repetition time was 5 ms. The accumulation time was 12–14 h.

#### Size Exclusion Chromatography

Size exclusion chromatography was performed at 5 °C using a Superdex 200 Increase 10/300 GL column in FPLC. Then 150 μL of 12.5 μM CPEB4* was injected using a 500 μL loop. The flow rate was 0.5 mL/min. The correlation between protein molecular weight and elution volume was determined using Bio-Rad’s Gel Filtration Standard kit ranging from 1.3 to 670 kDa.

## Results

### Macroscopic Behavior

**Figure 1:**
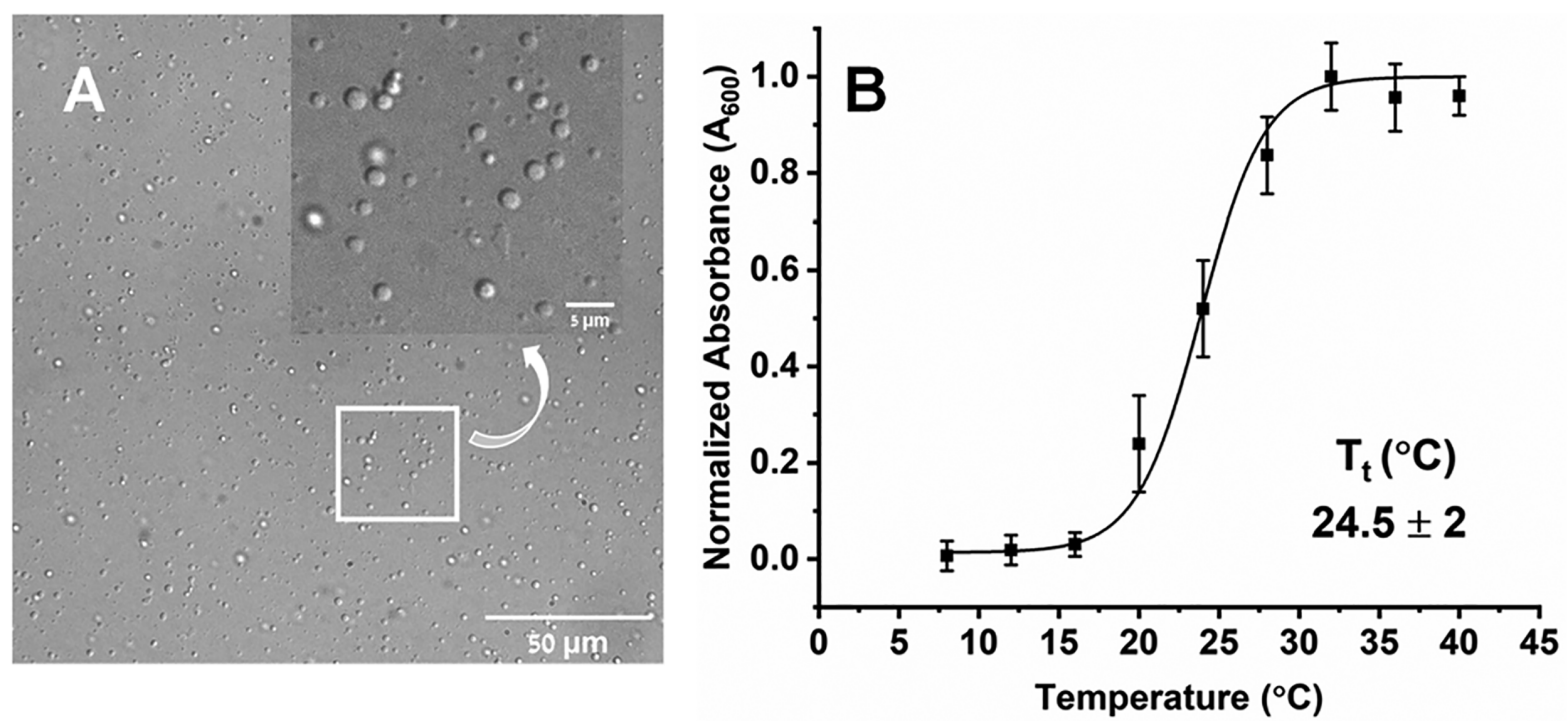
(A) RT microscope image of 10 μM CPEB4* in 100 mM NaCl. (B) Absorbance of 10 μM CPEB4* in 100 mM NaCl as a function of temperature and the corresponding fit to a sigmoidal curve.

We started with characterizing CPEB4* LLPS by optical microscopy where the behavior of CPEB4* was found to be similar to that of the corresponding nonlabeled CPEB4_NTD_ (Figures S2D and S3). At room temperature (RT) and in the presence of 100 mM NaCl (see Table S1 for buffer details), CPEB4* formed droplets with average diameters that scaled with protein concentrations; e.g., 1–2 μm for 10 μM and 10–15 μm for 96 μM, respectively (Figures 1A, and S3A–D). Upon cooling by incubating on ice, the droplets disassembled but they reformed in response to heating, and during the process smaller droplets fused and coalesced into larger droplets, thus confirming their liquid-like property (Figure S4). To determine the LLPS transition temperature, *T*_t_, we recorded either the bulk absorbance at 600 nm or followed the polydispersity index from DLS between 4 and 45 °C (Figure S5). For 10 μM CPEB4* we obtained a sigmoidal absorbance curve with *T*_t_ = 24.5 ± 2 °C (Figure 1B). Both microscopy and absorption results show that droplets form reversibly with increasing temperature, exhibiting a lower critical solution temperature (LCST) behavior. Because protein and salt concentrations are crucial parameters for LLPS,^27^ we examined their effect on *T*_t_ and found that both stabilize the droplets as *T*_t_ reduced with increasing protein and salt concentrations (Figure S5B–C). Control experiments in the absence of NaCl and under denaturing conditions, in the presence of guanidinium chloride (GdmCl, 3M), did not detect droplets by optical microscopy (Figure S3E–F).

### Molecular Level Behavior: Non-LLPS State

**Figure 2:**
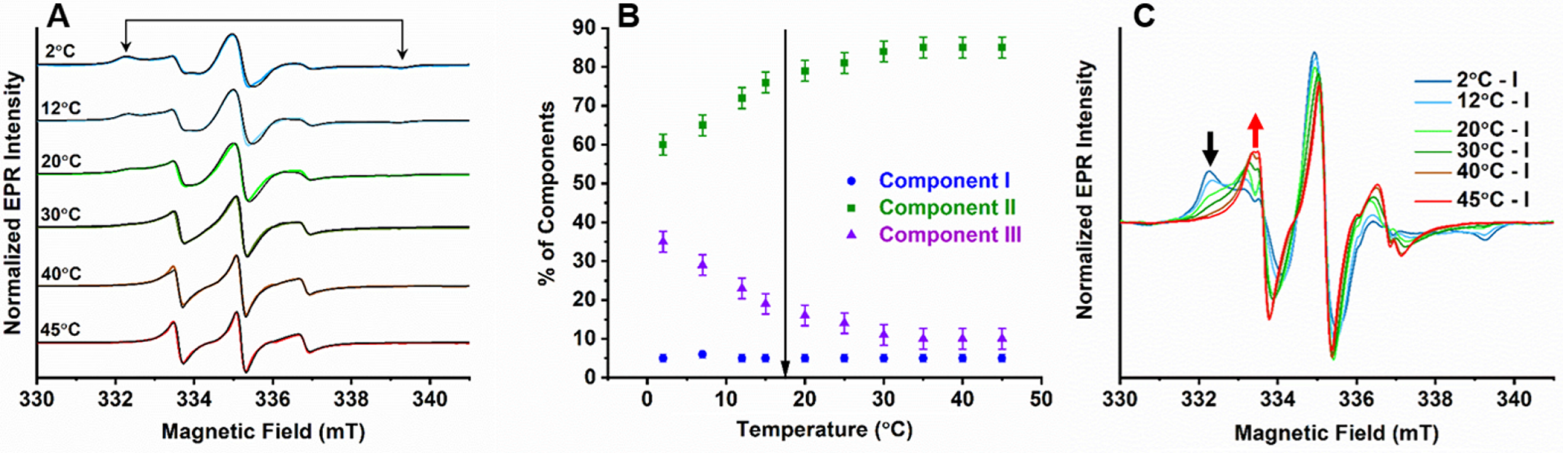
(A) Comparison of RT EPR spectra of denatured (28 μM, in 3 M GdmCl, black) and phosphorylated CPEB4* (20 μM, red.). The * marks a cavity background signal. For the phosphorylation experiments 3-malemide proxyl (MSL) was used as spin label. (B) EPR spectrum of CPEB4* (112 μM, 100 mM NaCl, pH 8) in a non-LLPS state (2 °C) and the corresponding simulations (black) with three components: a fast motion (**I**) in blue, an intermediate motion (**II**) in green, and a slow motion (**III**) in purple. Black and blue arrows indicate the characteristic features of slow and fast motion species, respectively. The simulation parameters are presented in Table S2. (C) W-band DEER data in logarithmic scale, measured at 25 K, of 80 μM CPEB4* in 3 M GdmCl (blue), 60 μM CPEB4* frozen after incubating at RT (LLPS, black), and frozen after incubating over ice (LT, non-LLPS, red). The corresponding straight lines represent a linear fit.

After characterizing the temperature-, salt- and concentration-dependent LLPS macroscopic behavior of CPEB4*, we set out to explore its dynamic properties under the same conditions using EPR spectroscopy. First we explored the EPR characteristics of CPEB4* under non-LLPS conditions. The RT EPR spectrum of phosphorylated CPEB4* (Figures 2A and S6), which does not phase separate,^36^ is practically identical with that of denatured CPEB4* in 3 M GdmCl, showing characteristic of a highly mobile nitroxide in Figure 2A, which is expected for IDP’s as CPEB4*.^44^ Surprisingly, the spectrum of unphosphorylated CPEB4* at 2 °C, which is well below the LLPS transition temperature, shown in Figure 2B, was very different than that of the phosphorylated CPEB4* (Figure S7) and exhibited clear features of multiple components, some of which are typical of a highly restricted motion. The outer extrema, indicated by black arrows in Figure 2B, give a separation of 7.02 mT, which is very close to the 2A_*zz*_ value (7.14 mT) determined from the W-band echo-detected EPR (EDEPR) spectrum of this sample at 25 K (Figure S8A) and therefore assigned to a practically rigid limit component. The presence of a component undergoing fast motion is obvious as well, and its features are indicated by blue arrows in Figure 2B. Using the Easyspin routine Chili,^39^ we tried to simulate the spectrum using two components, one undergoing fast motion with a correlation time, τ_c_ = 1.2 × 10^–9^ s and the second one featuring practically a rigid limit spectrum with τ_c_ > 1.6 × 10^–7^ s (Figure S8C–D), but we were unable to obtain a satisfactorily fit. Changing to the MOMD (microscopic order/macroscopic disorder) model with anisotropic rotational diffusion in combination with order parameters^45^ did not improve the fit. We also tried to fit the spectrum with a distribution of correlation times, which should be appropriate for an IDP, but the fit was still unsatisfactory. This suggested that a third component is present. We resolved the spectrum of the third component by subtracting the simulated rigid limit and fast motion components from the experimental spectrum (Figure S8E). Indeed, adding a third component with an intermediate motional regime (τ_c_ = 4.2 × 10^–9^ s), applying the isotropic rotational diffusion model, gave a very good fit as shown in Figure 2B. We realize that using an isotropic rotational diffusion model is an oversimplification of the system where the spin label should experience some local order and an ordering potential. In this case, the general MOMD model should be more appropriate. MOMD has been successfully applied to single component spectra of highly structured proteins like T4 lysozyme.^46^ Applying the MOMD model to simulate the slow and intermediate components would require fitting too many parameters, and it would be impractical to get any set of parameters that will be more meaningful than the simplified method we use for this work. Several reports in the literature have shown that EPR spectra of spin labeled IDPs can be simulated using the isotropic model^44,47,48^ and because CPEB4_NTD_ is an IDP we followed this approach. We use the simulations primarily as a tool to quantify the populations of the three components as a function of temperature, concentration and salt content. All simulation parameters are given in Tables S2 and S3. We assigned these three components as populations **I**, **II**, and **III** with spin-label undergoing fast, intermediate, and slow rotational diffusion and having relative abundances of 5%, 60%, and 35%, respectively. The spectral resolution and consequently the error range of the hyperfine parameters (see Table S2) did not allow drawing conclusions regarding differences in the polarity of the environment of the three species.

We assigned component **I** to monomeric native CPEB4* on the basis of its similar EPR spectrum to phosphorylated and denatured CPEB4*. We exclude the possibility that component **I** is a free spin label because its spectrum differs considerably from that of a free label, which comprises three sharp lines with equal intensities. The simplest explanation for the coexistence of CPEB4* with different dynamic properties would be the presence of three conformations of monomeric CPEB4*. However, the presence of a conformation experiencing a slow motion close to rigid limit in the non-LLPS state for an IDP with a spin label positioned at the C-terminal end seemed to us unlikely and suggested the presence of oligomerization. To substantiate this hypothesis we initially carried out CW-EPR measurements on a 1:5 spin-diluted sample, where the spin labeled protein was mixed with unlabeled protein (Figure S9).^48^ Such a dilution should decrease the intermolecular spin–spin distances in the oligomer and lead to narrowing of the EPR spectrum. However, we did not observe any narrowing of the spectra both at 2 °C (non-LLPS) and 20 °C (LLPS state). This observation is not surprising and does not necessarily contradict oligomerization due to the large size of the protein (448 amino acids) resulting in spin–spin distances that may be too large to be resolved in the CW-EPR spectra due to other broadening mechanisms such as distributions of hyperfine couplings.

Subsequently, we turned to pulse EPR measurements, which are more sensitive to spin–spin interactions than CW-EPR. We shock-froze the sample, precooled over ice, in liquid nitrogen cooled isopentane and recorded its echo decay and carried out DEER measurements. The results were compared to CPEB4* in GdmCl, which served as a reference for a homogeneous distribution of monomeric CPEB4* in a frozen solution. The echo decay of the non-LLPS state was distinctly much faster than in GdmCl (Figure S8B) indicating a much higher local concentration for the former. The DEER background decay is a function of the local spin concentration^49^ and can be applied to determine the local concentration^50−52^ and potential aggregation.^53^ The denatured CPEB4* showed the expected DEER exponential decay consistent with a 80 μM protein solution (Figure 2C), whereas the non-LLPS sample exhibited a remarkable increase in the DEER decays. This increase and the deviation from exponential decay indicate a high local concentration and provide experimental evidence for the presence of soluble CPEB4* assemblies in the non-LLPS state. We also noticed that the sample suffered a substantial loss in echo intensity due to very fast relaxation (Figure S8B). The weaker light scattering of the non-LLPS state in the absorbance and DLS data compared to the LLPS state (Figures 1B and S4) indicates that the size of these assemblies are much smaller than that of the droplets. Finally, we confirmed the presence of the assemblies by size exclusion chromatography measurements in the non-LLPS state (5 °C) (Figure S10). We thus conclude that prior to the LLPS state CPEB4* is present in three distinct dynamic and structural forms, a monomer undergoing fast local motions (component **I**), assemblies with component **II** with intermediate motion and component **III** experiencing slow motion.

### Molecular Level Behavior: LLPS State

**Figure 3:**
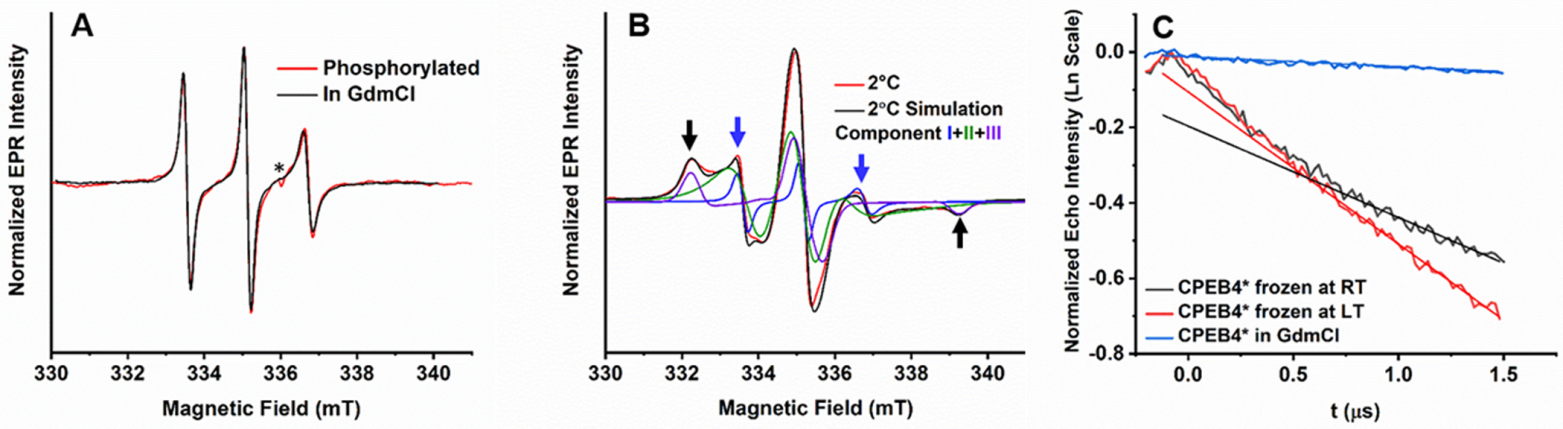
(A) Temperature dependent EPR spectra of CPEB4* (112 μM, 100 mM NaCl, pH 8) and the corresponding simulations (black). Arrows indicate signatures of the slow motion spectrum of component **III**. (B) Relative populations of components **I**, **II** and **III** as a function of temperature. The arrow indicates LLPS transition temperature (17.5 ± 2 °C). (C) Same EPR spectra as in part A of CPEB4* after subtracting the simulated component **I** spectrum. Black and red arrows mark the decay and rise of component **III** and **II**, respectively, with temperature.

To track changes in CPEB4* dynamics upon the onset of LLPS we recorded EPR spectra in the range of 2–45 °C, upon heating and cooling. The EPR spectra showed a reversible temperature dependence (Figure 3A, S11A–D). In the range of 2–20 °C, the spectra exhibit a clear decrease in the spectral signature component **III**; at the higher range, these signatures became less resolved. The decrease in the component **III** signature was associated with an increase in the signature of the component **II**. We simulated the EPR spectra over the entire temperature range using the three components discussed above, employing the isotropic rotational diffusion model. From the simulations we obtained the temperature dependence of the relative populations of **I**, **II**, and **III** (Figure 3B). Increasing the temperature from 2 to 45 °C, the relative abundance of components **II** increased from 60% to 85% and **III** decreased from 35% to 10% (Table S3). Surprisingly, the abundance of component **I** remained at 5% (Figure 3B) throughout the temperature range studied.

The simultaneous change in the population of **II** and **III** with increasing temperature suggests a temperature-dependent exchange between them. This exchange in population was particularly conspicuous when temperature dependent spectra were plotted after subtracting component **I** (Figure 3C). These spectra reveal a rather smooth change in the features of the EPR spectra, independent of the model used to simulate component **II** and **III**. A sigmoidal fit of the normalized populations of **II** and **III** versus temperature yielded an inflection point of 14.5 ± 1.5 °C (Figure S11E), which is close to the *T*_t_ value obtained from macroscopic LLPS measurements (*T*_t_ = 17.5 ± 2 °C, Figure S5C). However, the inflection was very broad, which is in contrast to the sharp LLPS transition, suggesting that it may not reflect the LLPS transition. The temperature dependence of τ_c_ also varied smoothly throughout the studied temperature range (Figure S12), thus being insensitive to the onset of LLPS. The EPR lineshapes and their temperature dependence show that the exchange rates between the various components are slow on the EPR time scale, and therefore the individual line shapes could be resolved.^54,55^ Unfortunately, we could not access the transition from monomeric CPEB4* to components **II** and **III** in the non-LLPS state as this probably takes place at concentrations well below the sensitivity limit of EPR. Finally, we did not observe any significant change in line shapes over a time of 20 h of keeping the sample at room temperature in the LLPS state.

To determine whether the observed dynamic properties are a function of the site of spin labeling, we generated an additional CPEB4_NTD_ construct, in which we mutated cysteine 441 to serine, and introduced an alternative cysteine at residue 320 (CPEB4_NTD_-C441S/G320C*), which is closer to the center of CPEB4_NTD_. The EPR spectra of CPEB4_NTD_-C441S/G320C* displayed same temperature dependent behavior as CPEB4*, indicating that both spin labels experience similar microenvironments (Figure S13) and that labeling positions did not influence the observed dynamics at least in the C-terminal region of CPEB4*.

After establishing the presence of three distinct dynamic components and their relation to LLPS, we proceeded to explore their distribution in the condensed (droplets) and dilute phases. To separate the dilute and condensed phases we sedimented the 112 μM CPEB4* solution at RT by centrifugation^56^ and recorded the EPR spectrum of the resulting supernatant (dilute phase). We found a single species with EPR characteristics similar to component **I** (Figure S8G), indicating that it constituted the primary species of the dilute phase in the LLPS state. From the simulation of the EPR spectrum, we have an estimation that 5% of the total CPEB4* would be in the dilute phase and the rest are in the condensed phase.

To examine the local concentration within the droplet, we carried out echo decay and DEER measurements on CPEB4* frozen from RT which is in the LLPS state. The DEER data (Figure 2C, black) showed an intense background decay, again deviating from exponential decay, indicating the presence of short intermolecular distances. This observation is also supported by the much faster echo decay, where spin–spin interactions dominate the phase relaxation (Figure S8B).^57^ The similar echo-decay rate and close DEER decays in the non-LLPS and LLPS states suggest the presence of small assemblies within the droplets, and it is their local concentration which determines the echo and the DEER decays.

### Effect of CPEB4* and Salt Concentrations

Our macroscopic measurements showed that both CPEB4* and NaCl concentrations affected LLPS by lowering *T*_t_. Accordingly, to correlate the observed **III** ⇌ **II** exchange with LLPS, we investigated the effect of [CPEB4*] and [NaCl] on the EPR spectra and the associated components **I**, **II**, and **III**. Once we determined the abundance of components **I**, **II**, and **III** as a function of temperature from the simulation of the EPR spectra, we calculated the relevant equilibrium constants *K*_II/I_ = [**II**]/[**I**], *K*_III/I_ = [**III**]/[**I**], and *K*_II/III_ = [**II**]/[**III**].

**Figure 4:**
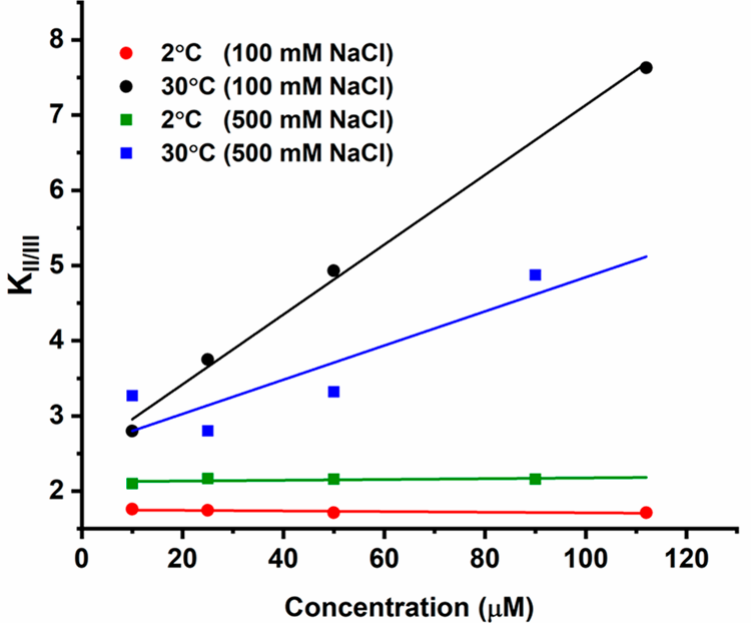
Dependence of *K*_II/III_ on [CPEB4*] at 2 °C (red) and 30 °C (black) for 100 mM (sphere) and 500 mM NaCl (square) at 2 °C (green) and 30 °C (blue).

We compared the temperature dependent EPR spectra of 112, 50, 25, and 10 μM of CPEB4* (Figure S14) and noted that the overall behavior was similar, but we identified some non-negligible differences in the lineshapes. From simulations of non-LLPS (2 °C) spectrum we detected an increase in τ_c_ of component **I** with increasing [CPEB4*] (Table S4) which might be a consequence of changes in the local viscosity. More interesting is the different variation of *K*_II/III_ with [CPEB4*] at 2 °C (below *T*_t_) and 30 °C (above *T*_t_). Figure 4 shows that while *K*_II/III_ is independent of [CPEB4*] in the non-LLPS state (see also Figure S14C-G), *K*_II/III_ increased linearly with [CPEB4*] in the LLPS state, indicating stabilization of component **II**. This provides a relation between the stabilization of the droplets with increasing [CPEB4*] and an increase in the population **II**, which is the dominant species in the droplet.

Next, we examined the effect of salt concentration. Surprisingly, in the absence of salt, where CPEB4* does not exhibit LLPS (Figure S3E), we clearly detected the presence of components **I**, **II**, and **III** from the EPR spectra, albeit with different populations compared to 100 mM NaCl (Figure S15A,B). Increasing [NaCl] from 100 to 500 mM further affected the relative population of the three components for 10–90 μM [CPEB4*], as manifested by the EPR lineshapes (Figure S16).

In Figure 4, we compared the *K*_II/III_ dependence on [CPEB4*] in the non-LLPS (at 2 °C) and the LLPS state (at 30 °C) for two different salt concentrations (100 and 500 mM NaCl) (see also Figure S17). In the non-LLPS state, *K*_II/III_ increased with [NaCl], indicating preference of **II** over **III** with increasing salt, but it remained invariant with increasing [CPEB4*], in agreement with the effect of [CPEB4*]. In the LLPS state, similar to the 100 mM NaCl *K*_II/III_ increased with [CPEB4*] also at 500 mM (Figures 4, S16G, and S17) but with a milder dependence and a general reduction in *K*_II/III_ compared to 100 mM NaCl. This shows a salt-induced preference of **III** over **II**, in contrast to the non-LLPS state. Considering that increasing [NaCl] from 100 to 500 mM stabilizes the LLPS state as manifested by the significant reduction in *T*_t_, the results presented in Figure 4 reveal a correlation between the three component equilibria and LLPS, where high [NaCl] alters the **III**/**II** equilibrium and stabilizes LLPS. Interestingly, component **I** remained 5–6% for all salt concentrations tested. Finally, we noted small changes of τ_c_ with increasing [NaCl] within the tested temperature range; a general increase for components **II** and a decrease for components **III** (Figure S12). The τ_c_ behavior was in general similar to the 100 mM NaCl sample (Figure S12).

**Figure 5:**
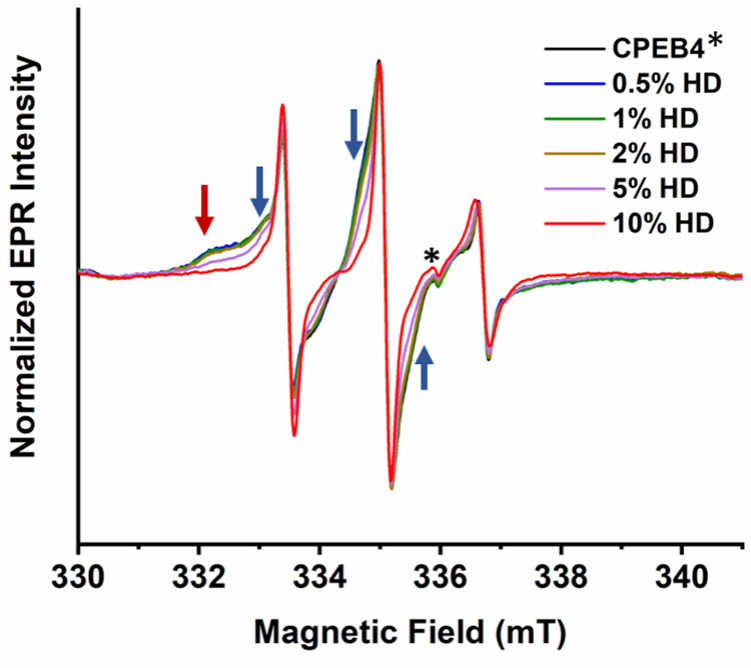
RT EPR spectra of 20 μM CPEB4* (100 mM NaCl, pH 8) in the presence of different percentage of 1,6-hexanediol (HD). The black spectrum is CPEB4* in the absence of any HD. The red and blue arrows point to spectral signatures of component **III** and **II**, respectively. * marks a cavity background signal.

F**i**nally, to resolve the intermolecular interactions that stabilizes the assemblies of components **II** and **III** in non-LLPS and LLPS states, we added to a solution of 20 μM CPEB4* in 100 mM NaCl varying amounts of 1,6 hexanediol (HD), which is known to disrupt weak hydrophobic interactions.^58^ The EPR spectra of these solutions, depicted in Figure 5, show that at RT (∼20 °C), which is within LLPS range, for up to 2% HD (w/v) the EPR spectra remained practically invariant. In contrast, at 5% HD there was a significant increase in the mobile fraction and reduction in the relative populations of components **II** and **III**. At 10%, their associated signals were practically undetectable, and the spectrum became similar to that of component **I** (Figure 5). In addition, at 10% HD, no droplets could be detected. The addition of 10% HD also affected the EPR spectrum recorded at 2 °C, i.e., in the non-LLPS state (Figure S18), albeit to a different extent. It increased the mobility of components **III** and **II**, but it did not abolish them. These results show that hydrophobic interactions play a major role in the formation of component **III** and **II** and of LLPS.

## Discussion

Using a combination of UV–vis absorption, DLS, and microscopy for macroscopic characterization and CW and pulse EPR spectroscopy for molecular level analysis, we tracked the dynamics of CPEB4* from non-LLPS to LLPS states. From the EPR results, it was evident that in both states CPEB4* is present in three dynamic forms but in different proportions. These are as follows. (i) The first is a flexible monomer, having low population and experiencing fast motion similar to that found when denatured or phosphorylated. In LLPS, it was found to reside within the dilute phase. (ii) The second form is small assemblies, which comprise component **II**, exhibiting intermediate motion, and component **III**, experiencing slow motion. We found the existence of these assemblies in CPEB4* concentrations of 10–112 μM. We have no experimental evidence regarding the structural features that distinguishes components **II** and **III**, except that they experience a different degree of restricted motion within an assembly of CPEB4* molecules. We hypothesize two options for the presence of components **II** and **III** in the assembly: (i) Components **III** and **II** are part of the same assembly, where component **III** represents a compact conformation of CPEB4* formed by strong intermolecular interactions at equilibrium with a less compact form assigned to component **II**. A possible picture would be component **III** situated at the core of an assembly and component **II** at the periphery. Alternatively, (ii) components **III** and **II** are part of different assemblies, one compact and other loose. We prefer option (i) because it is hard to rationalize two separate types of coexisting assembles over a broad temperature range. The intermolecular interactions creating the assembly are also responsible for the retardation of the motion of CPEB4*. Although there were subtle difference between the DEER traces recorded for non-LLPS and LLPS states, which requires further analysis, the current results implied high local spin concentration for both, namely these assemblies exist in both LLPS and non-LLPS states. The smooth temperature dependence, without any inflection, of τ_c_ over the non-LLPS and LLPS states also supports this conclusion. Thus, macroscopically CPEB4* is situated in two hierarchal environments of high local concentrations in the LLPS state: one is the small assemblies situated in the droplets, and the other is the droplets themselves.

**Scheme 1:**
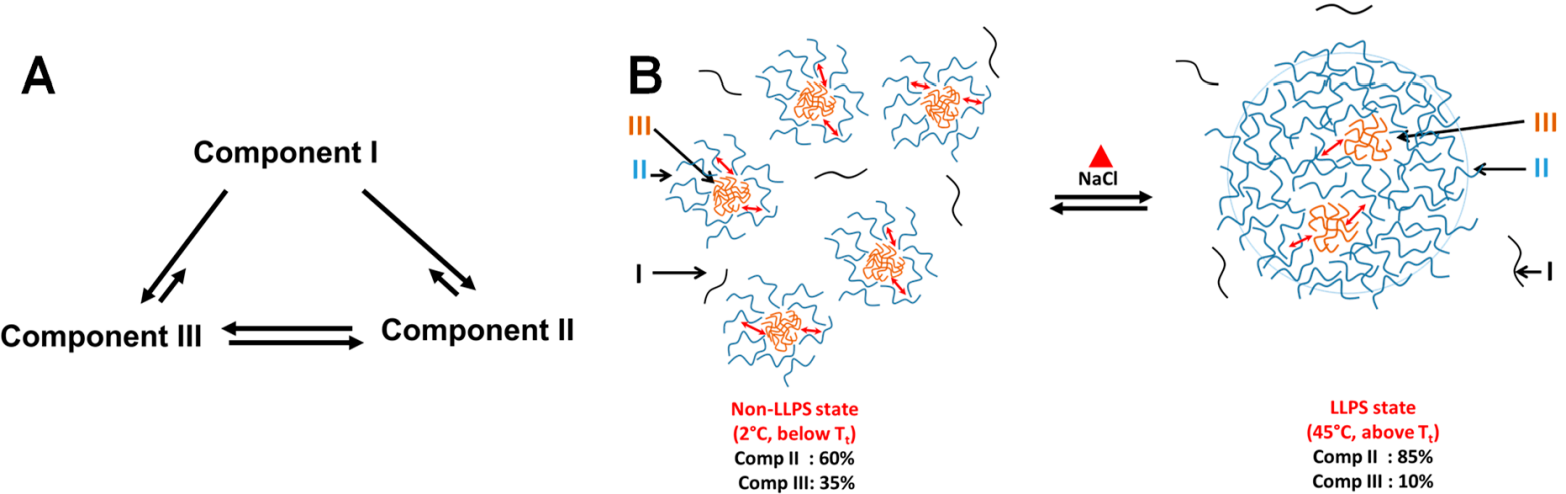
Suggested Model for LLPS Formation for CPEB4_NTD_ Key: (A) Equilibria between components **I**, **II** and **III**. (B) On the left, the non-LLPS state showing the presence of small assemblies containing compact and loose conformations. On the right hand side, droplets formed via interaction of loose conformations. Small red arrows in B, in both LLPS and non-LLPS states, indicate exchange between **II** and **III**, and the black arrows point to the corresponding forms. The schematic drawings are not to scale; the diameter of LLPS droplets ≫ the diameter of non-LLPS assemblies.

The constant and low population of component **I** across the LLPS strongly suggest that LLPS of CPEB4* does not proceed from the monomer, as has been observed for many LLPS forming proteins reported so far,^10^ but they evolve from preformed small assemblies of CPEB4* molecules. A similar unusual behavior was observed for a phosphorylated version of HP1α, nPhos-HP1α, which formed high-order oligomers in the non-LLPS state, and a correlation between the oligomers formation of LLPS tendency was reported.^59^ In Scheme 1, we present a hypothetic schematic model for the LLPS process of CPEB4_NTD_. At the heart of it, there is an equilibrium among the three components (Scheme 1A), existing both below and above *T*_t_. With increasing temperature, in the presence of salt, the **III**/**II** equilibrium shifts toward component **II**, which facilitates the transient interactions among small assemblies that are crucial for droplet formation (Scheme 1B). These interactions are however subtle and weaker than those responsible for holding the small assemblies together and therefore do not result in clear discontinuities in the correlation times and relative populations.

The question that arises is the relevance of components **II** and **III** to the formation of the LLPS state. Their absence after phosphorylation and HD addition, which abolish LLPS, show that they are relevant. Furthermore, the relation between the **II**/**III** equilibrium and the CPEB4* LLPS process is demonstrated by the effect of [CPEB4*] and [NaCl] on *K*_II/III_ and *T*_t_. First, we observed that *K*_II/III_ was invariant to [CPEB4*] below *T*_t_ but increased linearly above it, and this relates the increase in the population of component **II** with [CPEB4*] with the macroscopic observation of increased stability of the droplets with increasing [CPEB4*]. It shows that LLPS has an effect on the **III**/**II** equilibrium (or *vice versa*) and that component **II** most likely stabilizes LLPS (or *vice versa*). The different behavior of *K*_II/III_ in non-LLPS and LLPS states and the different effect of HD addition suggests that there is difference between the characters of CPEB4* assemblies in the droplets (above *T*_t_) and the CPEB4* assemblies below *T*_t_. This is also supported by the different trend of *K*_II/III_ in the presence of higher salt concentration.

The effect of salt on *K*_II/III_ is interesting and more complex. The **II**/**III** equilibrium takes place also in the absence of salt but droplets do not form. The presence of salt triggered LLPS, indicating that salt stimulates the CPEB4* interactions associated with LLPS formation. Increasing [NaCl] had a significant effect on *K*_II/III_. In non-LLPS it elevated *K*_II/III_ (higher % of component **II**), which indicates that the compact assembly of component **III** has some ionic interactions that becomes weaker in high [NaCL] thus reducing its stability. The conformation of component **II** is possibly governed via hydrophobic interaction and hence is preferred over compact component **III** at high [NaCl]. We hypothesize that while in component **III** the hydrophobic groups are buried, in the loose conformation of component **II** they are more exposed and accessible for intermolecular interactions that steer droplet formation under permissive temperature and salt conditions. Interestingly, in the LLPS state *K*_II/III_ decreases with increasing [NaCl], namely there is a higher abundance of component **III**, despite a stabilized LLPS state. This suggests that the increased salt facilitates LLPS transient interactions afforded by component **II** and thus compensates for its lower population relative to lower [NaCl].

LLPS behavior of CPEB4* is complex and involves a delicate interplay between charge and hydrophobic interactions. CPEB4_NTD_ has an overall charge of −4 (Figure S1) and the disappearance of component **III** and **II** upon phosphorylation suggests that electrostatic repulsions can disrupt LLPS and destabilize the assemblies in the non-LLPS state. CPEB4_NTD_ comprises a large number of hydrophobic residues (Table S5 and Figure S1) among them a substantial number of aromatic residues, 24 Phe, 6 Tyr, and 6 Trp. LLPS of CPEB4_NTD_ exhibits a LCST behavior, where droplets exist above *T*_t_(10,60−62) and previous studies have shown that LCST is driven by hydrophobic interactions and a dominant positive entropy term.^10,23,37,61−64^ Moreover, Phe and Tyr have been predicted^65^ and shown to play key role in the intermolecular interaction in LLPS,^5,37,11,15,61,66,67^ which includes transient interactions *via* intermolecular charge−π and π–π interactions. We hypothesize that these hydrophobic and aromatic interactions can lead to entropy driven LLPS of CPEB4_NTD_.^23,37,64^ This is supported by our observation that 10% HD, known to break weak hydrophobic interactions, prevented LLPS formation. Salt is known to alter the hydration of side chain amino acids and can enhance hydrophobic interactions.^58,68^ FUS and tau undergoing charge-driven LLPS at low [salt] were also found to exhibit hydrophobicity driven LLPS at high [salt].^23,58^ Hence, high [salt] can reduce *T*_t_ due to enhanced hydrophobic interaction, as also observed for CPEB4*. We thus conclude that higher [protein] favor LLPS by increasing population of species **II** whereas high [salt] favors LLPS by increasing hydrophobic interactions.

Thus far, the multicomponent properties of CPEB4* are unique among the few reports of protein LLPS behaviors studied by EPR. EPR analysis of TDP-43 LLPS revealed a superposition of two components, one experiencing a fast motion, similar to monomeric component **I** of CPEB4* and the second, with rigid/slow motion attributed to irreversible oligomerization of TDP-43.^25^ The study of a truncated version (187 residues) of the human amyloid protein tau showed that it underwent LLPS in the presence of RNA.^24^ A single fast motion component was identified, but no differences in the dynamics between RNA and non RNA-containing mixtures were found. Tau LLPS was shown to primarily depend on hydrophobic interactions at very high salt concentration (at 3–4 M) showing LCST. A dominant, rigid component was the main constituent of the condensed phase, attributed to a LLPS-driven amyloid form of tau.^23^ A very recent EPR study of FUS LLPS, where 0.5% agarose hydrogel was added to stabilize the droplets, reported very subtle differences between the dispersed phase and LLPS state at RT and revealed highly mobile FUS molecules. However, reduction to 5 °C, which enhances LLPS as FUS exhibits UCST (upper critical solution temperature) revealed the appearance of a rigid limit component, which was associated with FUS within the droplets.^26^ An NMR LLPS study of the elastic like polypeptide (ELP) mimicking tropoelastin, revealed LCST with two dynamic components. One, corresponding to a fast diffusing monomer in the dilute phase, the other to a 100 fold slower diffusing monomer in the condensed phase.^62^ A relevant question is why two types of dynamic forms of CPEB4* (compact/**III** and loose/**II**) would exist in bimolecular condensates? A recent review has shown that the compact conformation with strong interaction can provide structural specificity whereas the loose form can quickly break/make weak interactions providing dynamical properties required for liquid droplets.^69^ The right balance between compact and loose forms can produce optimum functionality via structural hierarchy. However, it is currently hard to correlate CPEB4_NTD_ LLPS formation with function as it is not the full length protein. The structural features of these assemblies and their existence in the full length protein are yet to be determined.

## Conclusions

Using EPR spectroscopy, we resolved three populations with distinct dynamic properties, components **I**, **II**, and **III** which coexist both in non-LLPS and LLPS states of spin-labeled CPEB4_NTD_ (CPEB4*). These components are interconnected by three equilibria. Component **I** was assigned to a highly flexible, monomeric CPEB4* and had very little abundance below and above *T*_t_. Above *T*_t_, it is the major constituent of the dilute phase. Components **II** and **III** reside within soluble assemblies of CPEB4_NTD_ molecules, with component **III** likely situated in more compact region of the assembly as compared to component **II**. LLPS of CPEB4_NTD_ occurred with increasing temperature from a mixture of these three components and was associated with a shift of the **III** ⇌ **II** equilibrium toward component **II**. While no clear discontinuity in the correlation times, nor in the relative populations, was observed with temperature, our results from the salt and CPEB4* concentration effects on LLPS indicate that component **II** favors LLPS. Furthermore, the interactions leading to LLPS are primarily hydrophobic. Finally, LLPS of CPEB4* evolves from pre-existing soluble molecular assemblies, which differs from most systems undergoing LLPS reported so far.

## Supporting Information Available

Microscope images, absorption data, size exclusion chromatography data, EasySpin simulation and parameters, additional EPR data, EPR data at different salt and protein concentrations, and protein sequence and residues (PDF)
