# Correction to “Evolution of CPEB4 Dynamics Across Its Liquid–Liquid Phase Separation Transition”

**DOI:** 10.1021/acs.jpcb.1c10242

**Published:** 2021-12-14

**Authors:** Manas Seal, Chandrima Jash, Reeba Susan Jacob, Akiva Feintuch, Yair Shalom Harel, Shira Albeck, Tamar Unger, Daniella Goldfarb

The original article was published with error in the figures where Figures 2 and 3 were exchanged by the production house after the galley correction. Here we show correct Figures 2 and 3.

**Figure 2:**
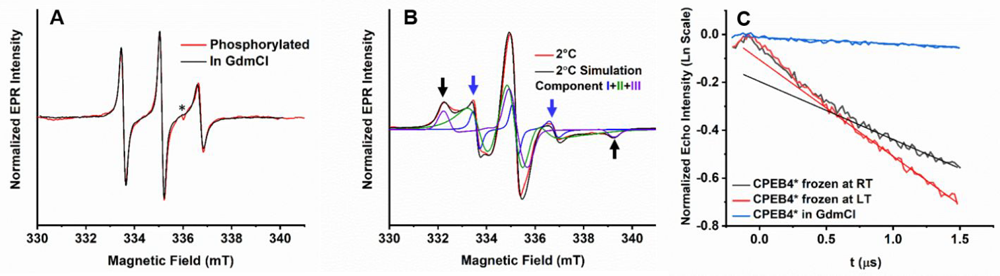
(A) Comparison of RT EPR spectra of denatured (28 μM, in 3 M GdmCl, black) and phosphorylated CPEB4* (20 μM, red). The * marks a cavity background signal. For the phosphorylation experiments 3-malemide proxyl (MSL) was used as spin label. (B) EPR spectrum of CPEB4* (112 μM, 100 mM NaCl, pH 8) in a non-LLPS state (2 °C) and the corresponding simulations (black) with three components: a fast motion (**I**) in blue, an intermediate motion (**II**) in green, and a slow motion (**III**) in purple. Black and blue arrows indicate the characteristic features of slow and fast motion species, respectively. The simulation parameters are presented in Table S2. (C) W-band DEER data in logarithmic scale, measured at 25 K, of 80 μM CPEB4* in 3 M GdmCl (blue), 60 μM CPEB4* frozen after incubating at RT (LLPS, black), and frozen after incubating over ice (LT, non-LLPS, red). The corresponding straight lines represent a linear fit.

**Figure 3:**
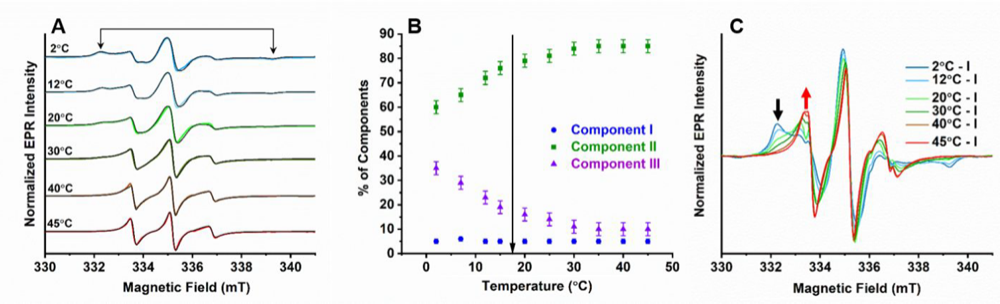
(A) Temperature dependent EPR spectra of CPEB4* (112 μM, 100 mM NaCl, pH 8) and the corresponding simulations (black). Arrows indicate signatures of the slow motion spectrum of component **III**. (B) Relative populations of components **I**, **II**, and **III** as a function of temperature. The arrow indicates LLPS transition temperature (17.5 ± 2 °C). (C) Same EPR spectra as in part A of CPEB4* after subtracting the simulated component **I** spectrum. Black and red arrows mark the decay and rise of component **III** and **II**, respectively, with temperature.

