# Supplementary material for: Evolution of CPEB4 Dynamics Across its Liquid–Liquid Phase Separation Transition
Source: J Phys Chem B. 2021 Nov 17;125(47):12947–57. doi: 10.1021/acs.jpcb.1c06696 (PMC8647080; doi:10.1021/acs.jpcb.1c06696)
Supplement: Supplementary file 1 — jp1c06696_si_001.pdf [file jp1c06696_si_001.pdf]

Supporting Information

Evolution of CPEB4 dynamics across its liquid-liquid phase separation transition

Manas Seal[1], Chandrima Jash[1], Reeba Susan Jacob[2], Akiva Feintuch[1], Yair Shalom Harel[3], Shira Albeck[4], Tamar Unger[4], Daniella Goldfarb[1]*

[1]Department of Chemical and Biological Physics, [2]Department of Biological Regulation, [3]Department of Structural Biology, [4]Department of Life Sciences Core Facilities, Weizmann Institute of Science, Rehovot, Israel

Contents

1. **$CPEB4_{NTD}$ Amino Acid Sequence** S2
2. **Effect of glycerol, labelling, temperature, protein and salt concentrations.** S4
3. **Phosphorylation of CPEB4* in Mass spectrometry** S8
4. **Simulation of the EPR spectra and pulse EPR results** S10
5. **CW-EPR under spin dilution** S12
6. **Size Exclusion Chromatography** S13
7. **EPR spectroscopy: Temperature dependence** S14
8. **EPR Spectroscopy: Effect of concentration** S17
9. **EPR spectroscopy: Salt effect** S18
10. **Interaction with 1,6 Hexanediol** S21

1. CPEB4$_{NTD}$ Amino Acid Sequence

A) CPEB4$_{NTD}$ Amino Acid Sequence (448 residues):

MGDYGFGVLV QSNTGNKSAF PVRFHPHLQP PHHHQNATPSPAAFINNNTA 50

ANGSSAGSAW LFPAPATHNI QDEILGSEKA KSQQQEQQDP LEKQQLSPSP 100

GQEAGILPET EKAKSEENQG DNSSENGNGK EKIRIESPVL TGFDYQEATG 150

LGTSTQPLTS SASSLTGFSN WSAAIAPSSS TIINEDASFF HQGGVPAASA 200

NNGALLFQNF PHHVSPGFGG SFSPQIGPLS QHHPHHPHFQ HHHSQHQQQR 250

RSPASPHPPP FTHRNAAFNQ LPHLANNLNK PPSPWSSYQS PSPTPSSSWS 300

PGGGGYGGWG GSQGRDHRR**G** LNGGITPLNS ISPLKKNFAS NHIQLQKYAR 350

PSSAFAPKSW MEDSLNRADN IFPFPDRPRT FDMHSLESSL IDIMRAENDT 400

IKARTYGRRR GQSSLFPMED GFLDDGRGDQ PLHSGLGSPH **C**FSHQNGE 448

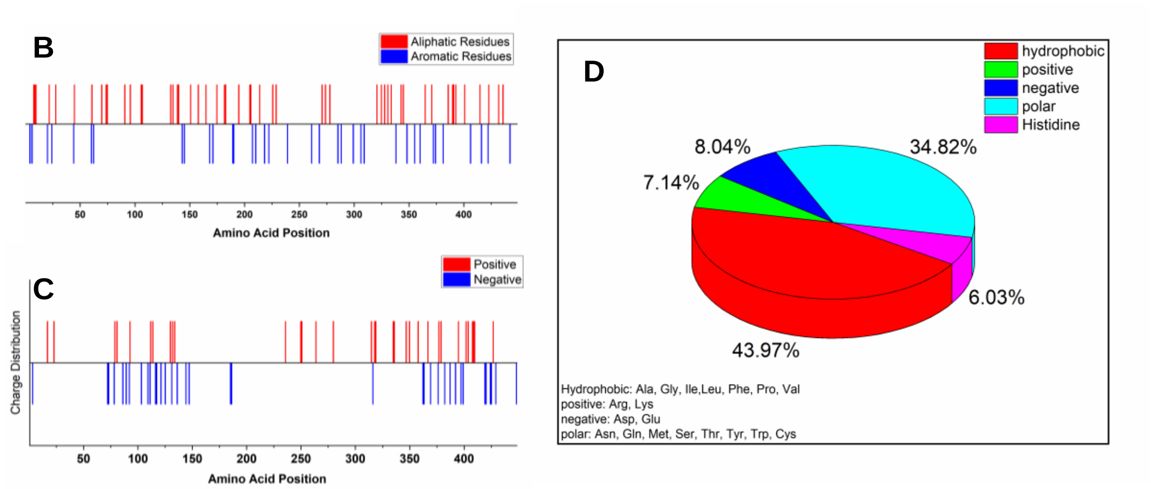

Figure S1. A) Amino acid sequence of CPEB4$_{NTD}$ (aa 1-448) and the labelling positions used in this study are highlighted in red, C441 and G320. Aliphatic and aromatic side chains (excluding His) of CPEB4$_{NTD}$ are shown in B) and positive and negative charged residues (excluding His) are in C) (obtained using EMBOSS Pepinfo). D) Chart representing the content of different types of amino acids in percentage.

Table S1. Details of the buffers used in the study.

Buffer	Buffer composition for purification
Lysis Buffer	50 mM TrisHCl pH 8, 100 mM NaCl, 0.5% TritonX-100, 1.4 mM beta marcapto ethanol (BME), protease inhibitor cocktail (PIC)
IB wash I	25 mM TrisHCl pH8, 0.5% TritonX 100, 1.4 mM BME, PIC, DNAse
IB wash-II	25 mM TrisHCl, pH 8, 1 M NaCl, 1.4 mM BME, PIC
IB re-solubilization	25 mM TrisHCl pH8.0, 100 mM NaCl, 8 M urea, 1.4 mM BME
Ni bufferA	25 mM TrisHCl pH8, 50 mM NaCl, 8 M urea, 20 mM imidazole, 1.4 mM BME
Ni buffer B	25 mM TrisHCl pH8, 50 mM NaCl, 8 M urea, 500 mM imidazole, 1.4 mM BME
Size exclusion buffer	25 mM Tris-HCl pH8, 3M Guanidinium chloride (GdmCl), 1mM Tris(2-carboxyethyl)phosphine hydrochloride (TCEP) and 0.05% NaN3
	**Buffer composition for experiments with labelled $CPEB4_{NTD}$**
Buffer for LLPS	25 mM Tris-HCl pH8, 100 mM NaCl
Buffer for denatured $CPEB4_{NTD}$	25 mM Tris-HCl pH8, 3M GdmCl

2. Effect of glycerol, labelling, temperature, protein and salt concentrations.

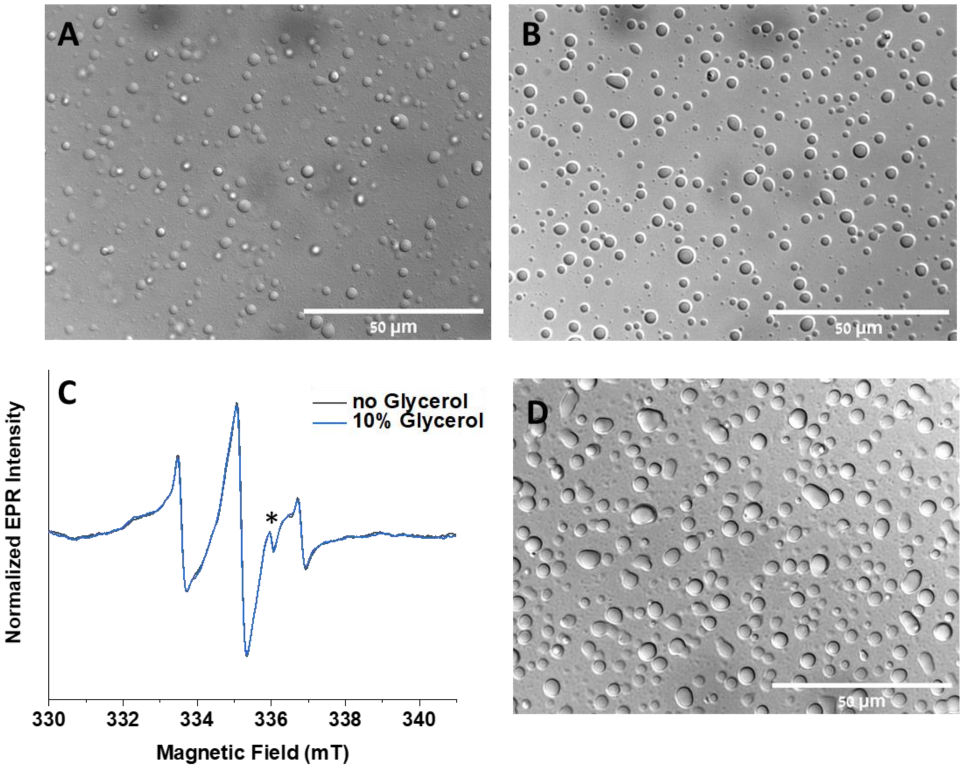

Figure S2. A) RT microscope image of 20 µM CPEB4* and in the presence of 10% glycerol in B). C) RT EPR spectra of the samples in A) and B). * marks a cavity background signal. D) RT microscope image of 50 µM unlabeled CPEB4$_{NTD}$. The buffer used for all the samples was 25 mM TrisHCl, pH 8, 100 mM NaCl. The scale bar is 50 µm.

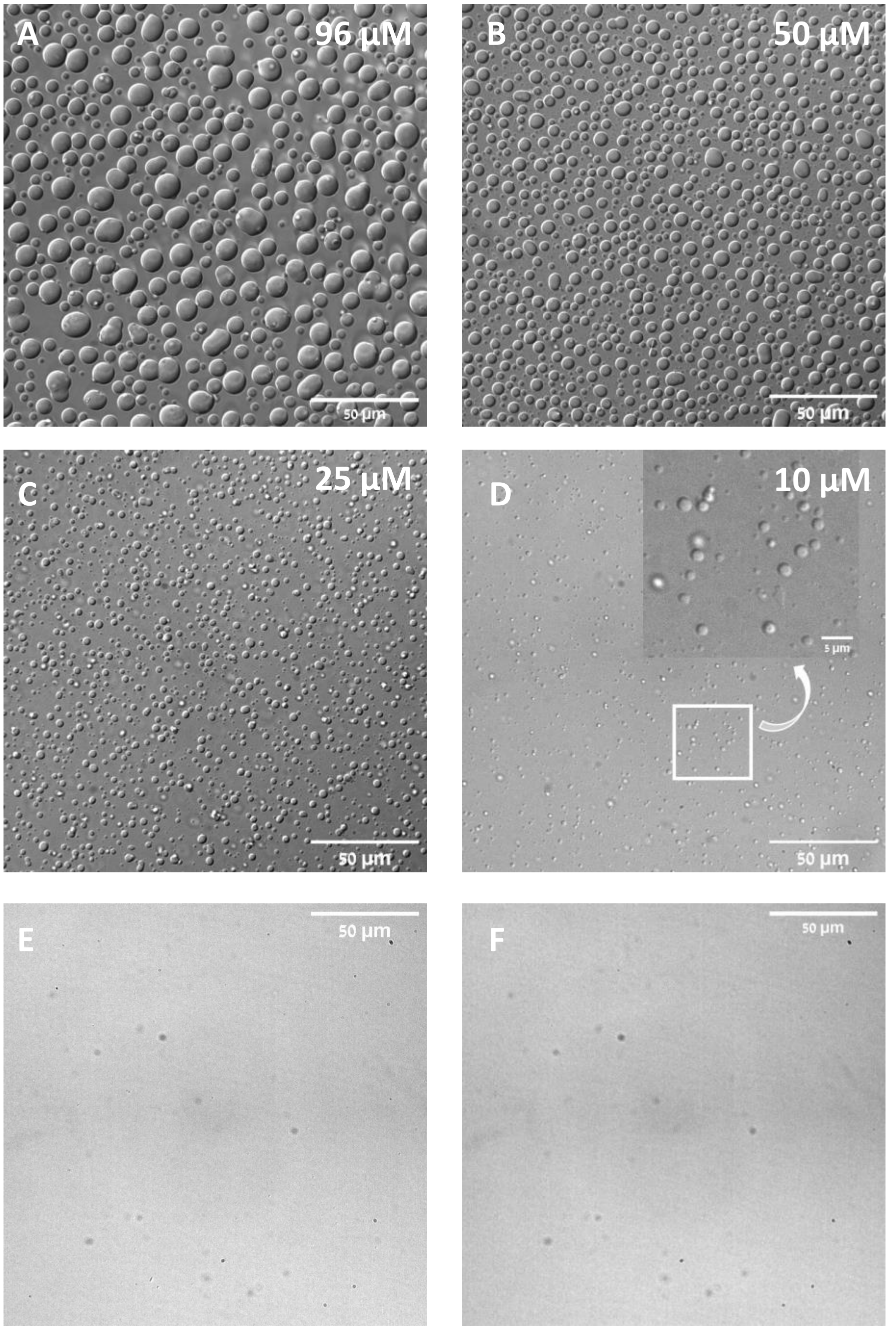

Figure S3. RT microscope image of CPEB4* solutions of A) 96 μM B) 50 μM C) 25 μM and D) 10 μM in 100 mM NaCl. E) 10 μM CPEB4* in the absence of salt and F) in the presence of 3M GdmCl, showing no droplets (black spots are from the background). The buffer used for all the samples were 25 mM TrisHCl, pH 8, 100 mM NaCl. The scale bar is 50 μm.

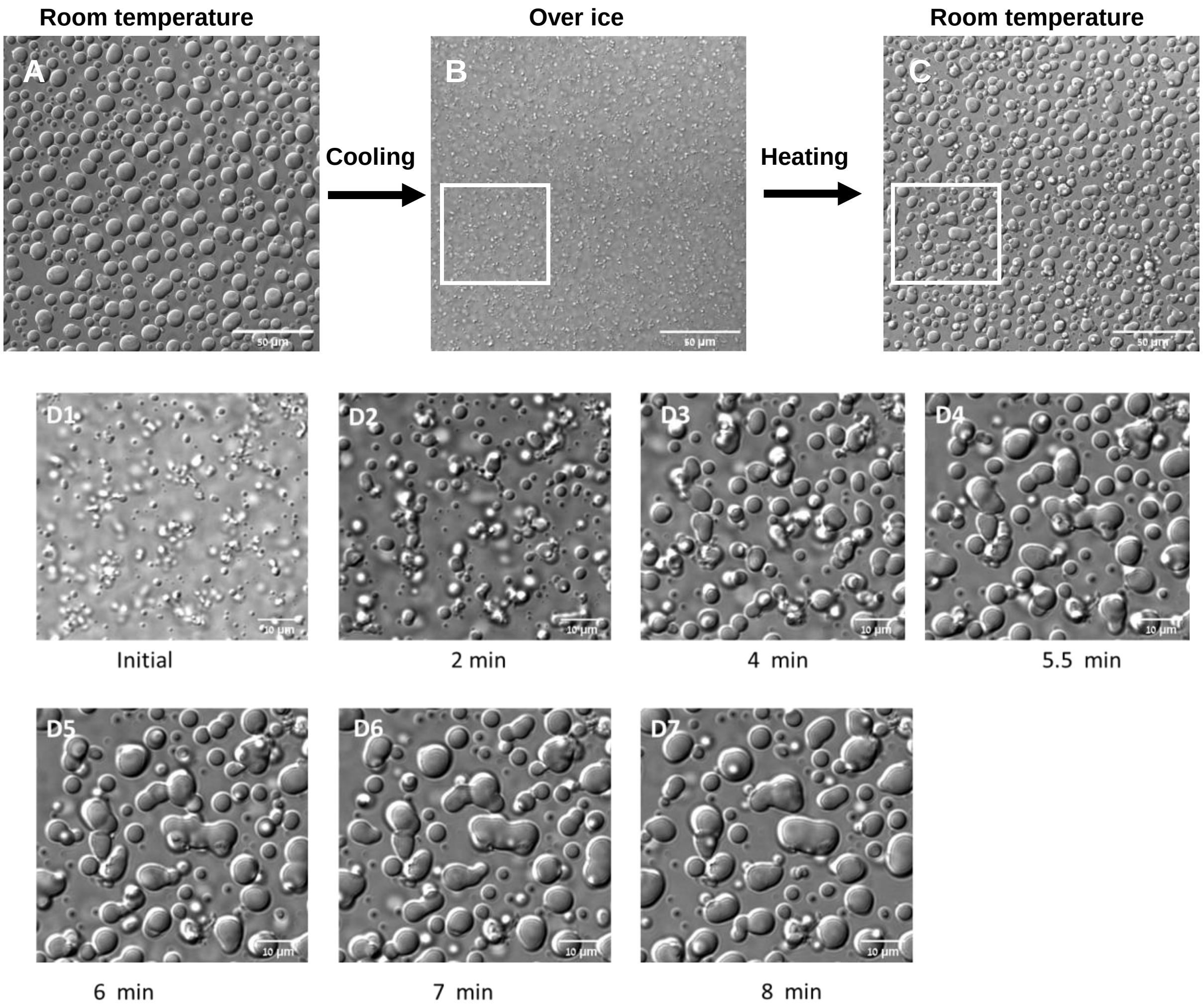

Figure S4. Microscope images of 96 μM CPEB4* in 100 mM NaCl, A) at RT. B) Same sample after cooling over ice for 15 minutes and C) when warmed to RT. The bar scale in A, B and C is 50 μM. D1-D8) Microscope images during sample warming to RT. D1 is the magnification of the selected area in B) and D7 is the magnification of the selected area in C). D2-D6 are at different times between D1 and D7. The small droplets in the images in B and D1 could be a result of delay in transferring the slide and focusing. The scale bar in D1-D7 is 10 μm.

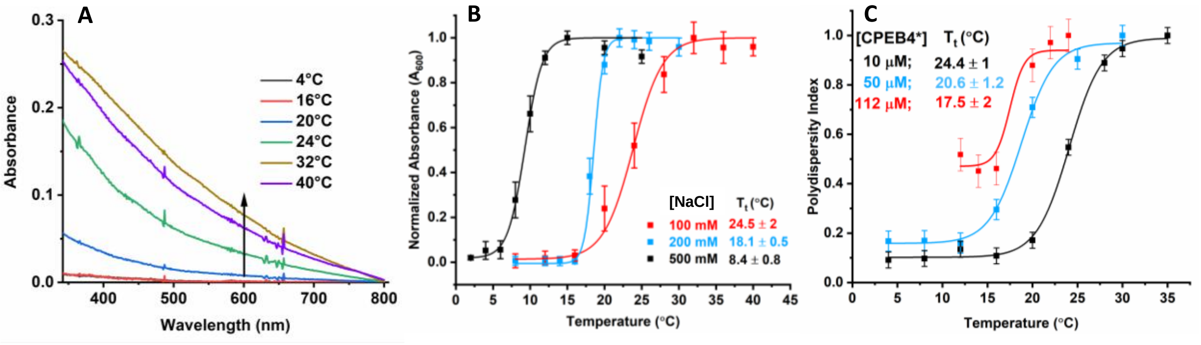

Figure S5. A) Absorption spectra of 10 μM CPEB4* in 100 mM NaCl at different temperatures. B) Absorbance at 600 nm of 10 μM CPEB4* in 100 (red), 200 (cyan) and 500 (black) mM NaCl as a function of temperature and the corresponding fit to a sigmoidal function. B) Plot of the polydispersity index vs temperature (°C) for 112 μM (red), 50 μM (cyan) and 10 μM (black) CPEB4* obtained from dynamic light scattering. The transition temperature determined from these fits are noted on the figure.

3. Phosphorylation of CPEB4* in Mass spectrometry

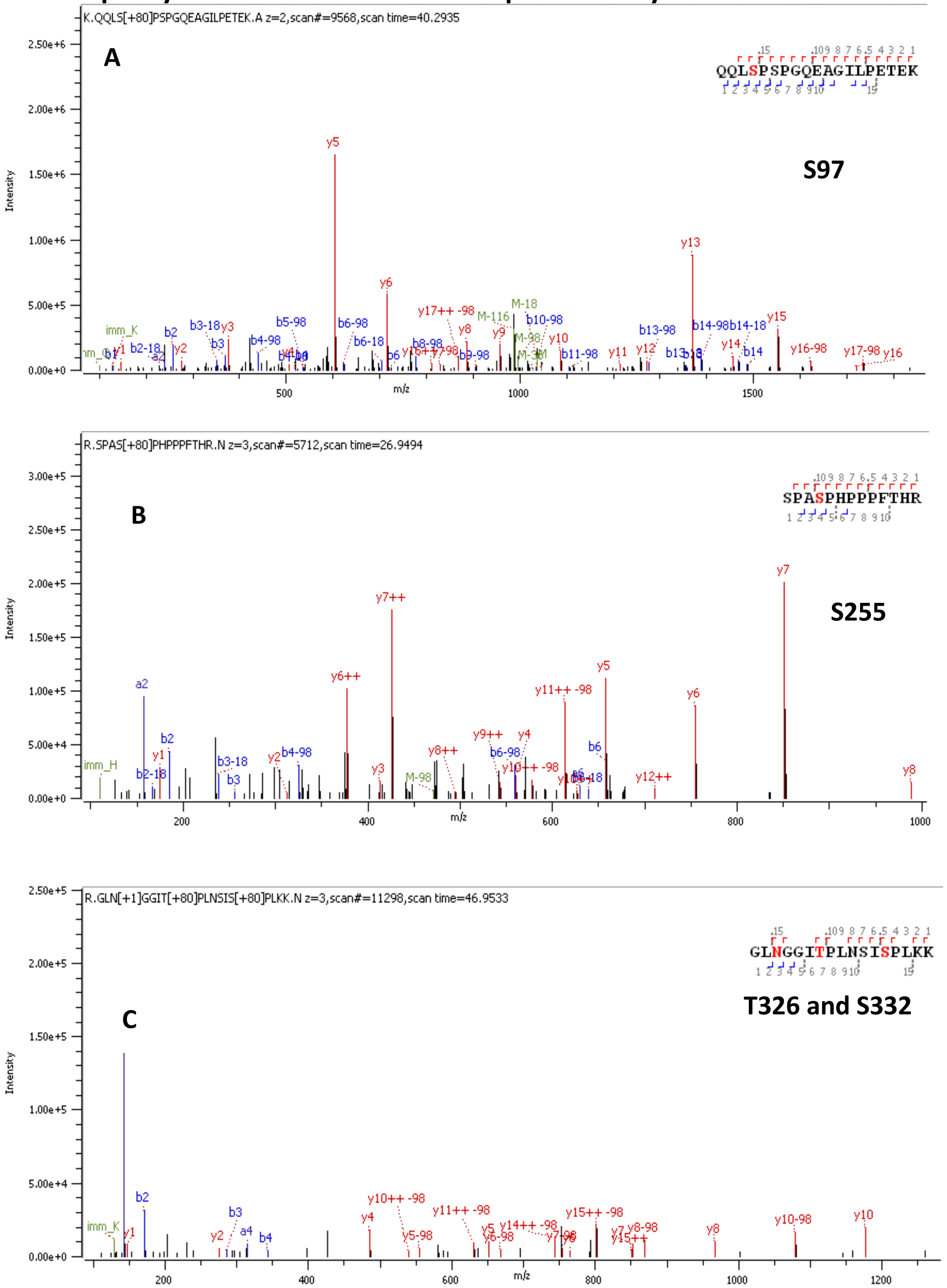

Figure S6. Mass spectrometry data for the phosphorylation sites at A) S97, B) S255 and C) S332 and T326 using Byonic search engine.

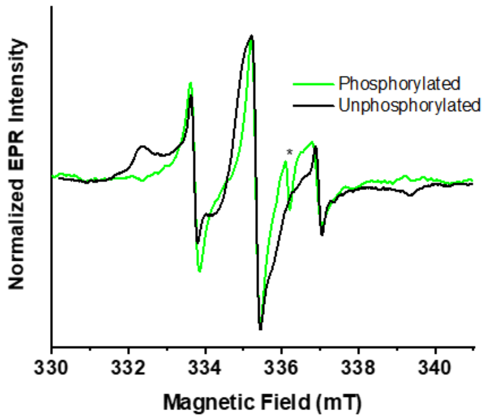

Figure S7. EPR spectra of 25 µM phosphorylated and unphosphorylated CPEB4* at 2°C. For the phosphorylation experiments 3-malemide proxyl (MSL) was used as spin label. * marks a cavity background signal.

4. Simulation of the EPR spectra and pulse EPR results

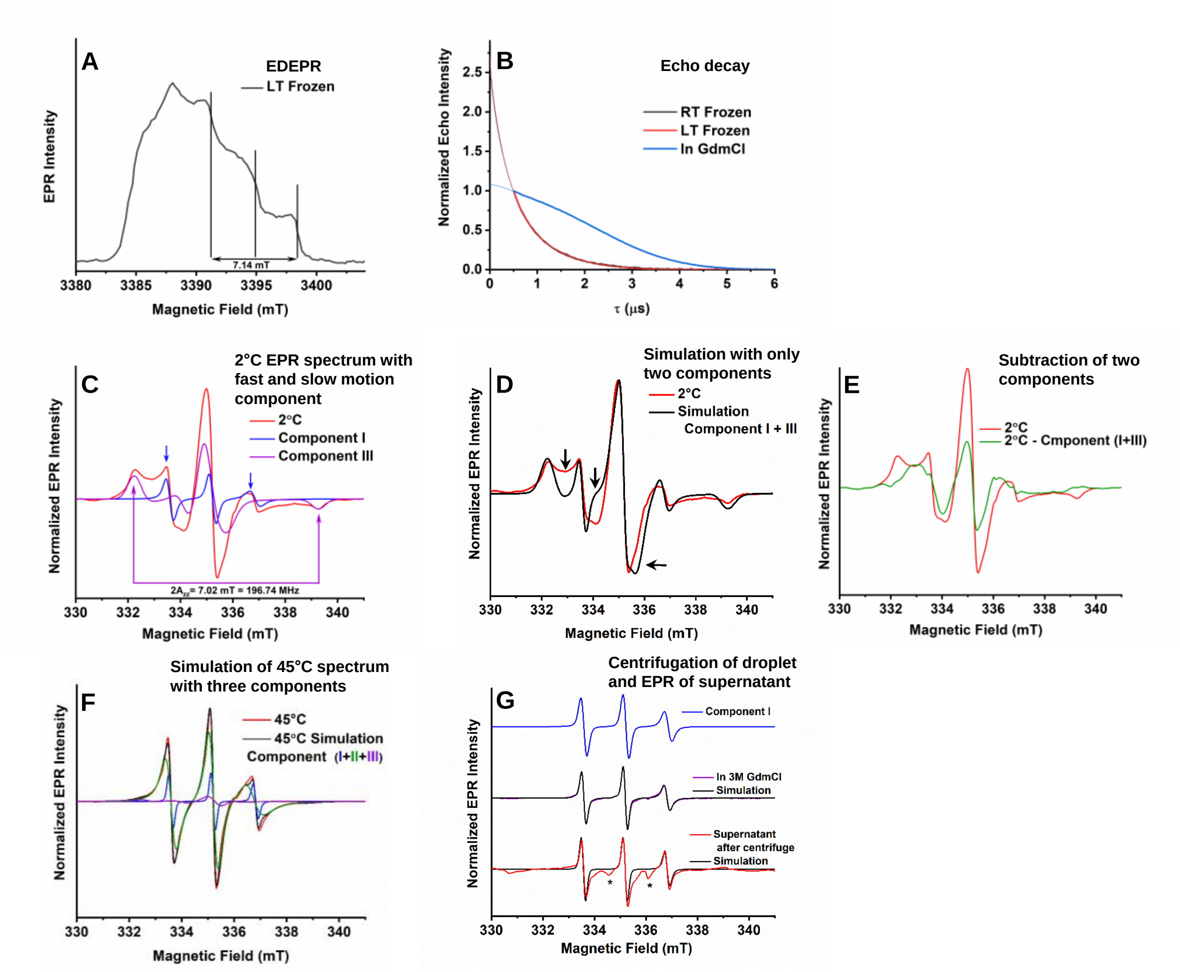

Figure S8. A) W-band EDEPR spectrum (25K) of non-LLPS state of CPEB4*. B) Echo decay (25K) of 80 μM CPEB4* in 3M GdmCl (blue), 60 μM CPEB4* droplet frozen from RT (black) and frozen from incubation over ice (red). The echo decay (bold line), normalized at τ= 0.5 μs, were fitted and extrapolated to t=0, by fitting to a stretched exponent (thin line). C) EPR spectrum of 112 μM CPEB4 at 2°C, simulated with component **I** (blue) and component **III** (purple). The arrows point to the respective components. D) The sum of simulated component **I** and **III** compared to the experimental spectrum where the arrows show regions of missing signal. E) Subtraction of both component **I** and **III** from the 2°C experimental EPR spectrum. F) EPR spectrum of the same sample at 45°C and simulation with three components. G) RT EPR spectrum of supernatant (red) after centrifuging 112 μM CPEB4* in 100 mM NaCl compared with simulated spectrum of component **I** (black). This simulation is shown also individually in blue. The purple spectrum corresponds to denatured of CPEB4* (in 3 M GdmCl) compared with component **I** simulations (black). The simulation parameters are given in Table S2. * marks a cavity background signal.

Table S2. *g* , ^{14}N hyperfine coupling values and linewidth parameters used in the simulations of the EPR spectra.

Simulation Parameters at 2°C	Components		
	**I**	**II**	**III**
**[g_x g_y g_z]**	2.0081± 0.0001<br>2.0061± 0.0001<br>2.0029 ± 0.0001	2.0081± 0.0001<br>2.0061± 0.0001<br>2.0029 ± 0.0001	2.0081± 0.0001<br>2.0061± 0.0001<br>2.0024 ± 0.0001
**[A_x A_y A_z] in mT**	0.67 ± 0.044<br>0.67 ± 0.044<br>3.59 ± 0.085	0.67 ± 0.044<br>0.67 ± 0.044<br>3.6 ± 0.07	[0.67 ± 0.044<br>0.67 ± 0.044<br>3.58 ± 0.035]
**Log(τ_c)**	-9 ± 0.062	-8.35 ± 0.03	-6.865± 0.065
**Line width (mT)**	0.215 ± 0.015	0.56 ± 0.04	0.525± 0.025
**% of components**	5 ± 0.5	62.5 ± 2.7	32.5 ± 2.6
**Simulation Parameters at 45°C**	**I**	**II**	**III**
**[g_x g_y g_z]**	2.0081± 0.0001<br>2.0061± 0.0001<br>2.0029 ± 0.0001	2.0081± 0.0001<br>2.0061± 0.0001<br>2.0029 ± 0.0001	2.0081± 0.0001<br>2.0061± 0.0001<br>2.0024 ± 0.0001
**[A_x A_y A_z] in mT**	0.67 ± 0.044<br>0.67 ± 0.044<br>3.55 ± 0.054	0.67 ± 0.044<br>0.67 ± 0.044<br>3.55 ± 0.054	[0.71 0.71 3.49]
**Log(τ_c)**	-9.675 ± 0.075	-8.735± 0.085	-7.8
**Line width (mT)**	0.19± 0.01	0.18 ± 0.02	0.4
**% of components**	5 ± 1	85 ± 4	10 ± 2.5

Table S3. Relative populations and the corresponding τ_c values of component **I**, **II** and **III** obtained from simulations of the 112 μM CPEB4* EPR spectra (2-45°C) shown in Figure 2B.[a]

Temperature (°C)	Component I		Component II		Component III		Temperature (°C)	Component I		Component II		Component III	
	%	log(τ_c)	%	log(τ_c)	%	log(τ_c)		%	log(τ_c)	%	log(τ_c)	%	log(τ_c)
**2**	5	-8.95	60	-8.37	35	-6.8	**25**	5	-9.25	81	-8.53	14	-7.5
**7**	6	-8.95	65	-8.38	29	-7.0	**30**	5	-9.45	84	-8.58	11	-7.55
**12**	5	-9.05	72	-8.35	23	-7.1	**35**	5	-9.45	85	-8.66	10	-7.6
**15**	5	-9.10	76	-8.4	19	-7.2	**40**	5	-9.55	85	-8.72	10	-7.7
**20**	5	-9.15	79	-8.5	16	-7.3	**45**	5	-9.60	85	-8.82	10	-7.8

[a] The line width for the simulation of component II decreased with increasing temperature. It changed from 0.6 mT at 2°C to 0.4 mT at 20°C to 0.2 mT at 45°C.

5. CW-EPR under spin dilution

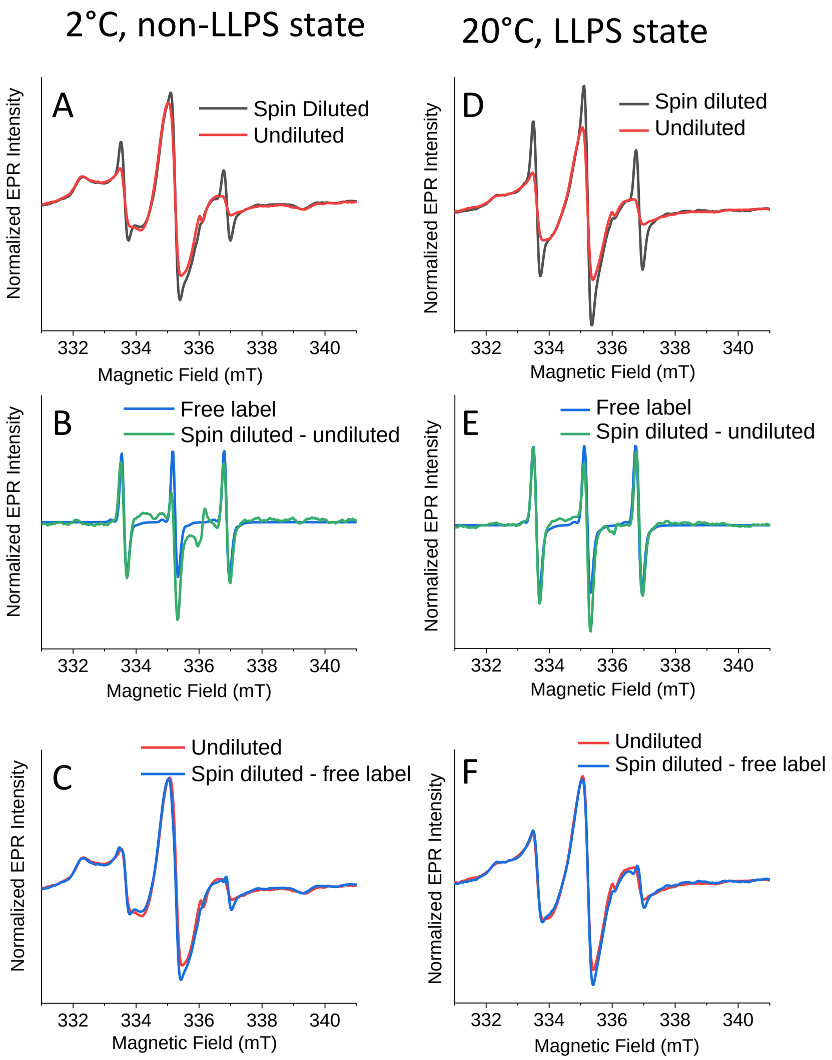

Figure S9. A) CW EPR spectra of spin diluted sample (15 µM spin labeled out of 70 µM total protein concentration, black) and undiluted sample (70 µM spin labeled protein, red) at 2°C. B) Subtraction of undiluted sample from the spin diluted sample (after normalization to component **III** (green) and comparison with free spin label (blue). This shows that the difference between the spectra in A is due to free spin label in the diluted sample, which is a consequence of residual DTT in the unlabeled protein. C) Subtraction of the spectrum of free spin label from spin diluted sample and comparison with undiluted sample showing that both are same. D), E) and F) are the same A), B) and C) but at 20 °C.

6. Size Exclusion Chromatography

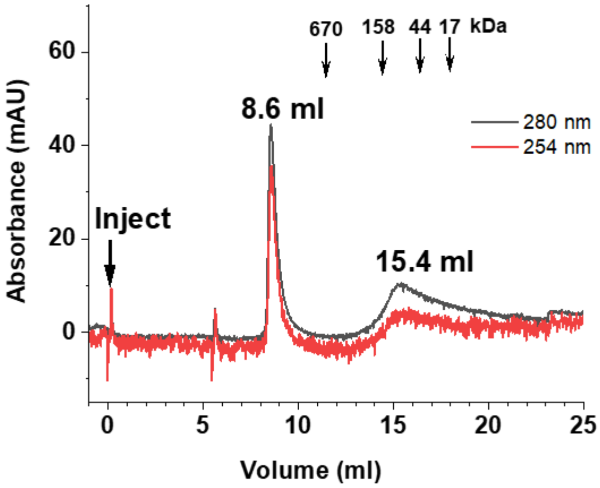

Figure S10. Size exclusion chromatogram of CPEB4* at 5°C using Superdex 200 Increase 10/300 GL column. Injected amount: 150 µL of 12.5 µM CPEB4*. The arrows indicates elution position for molecular weight markers from standard kit. The sharp peak at 8.6 ml indicates the presence of assemblies of CPEB4* molecules. The broad peak at 15.4 ml could be attributed to monomers of CPEB4*.

7. EPR spectroscopy: Temperature dependence

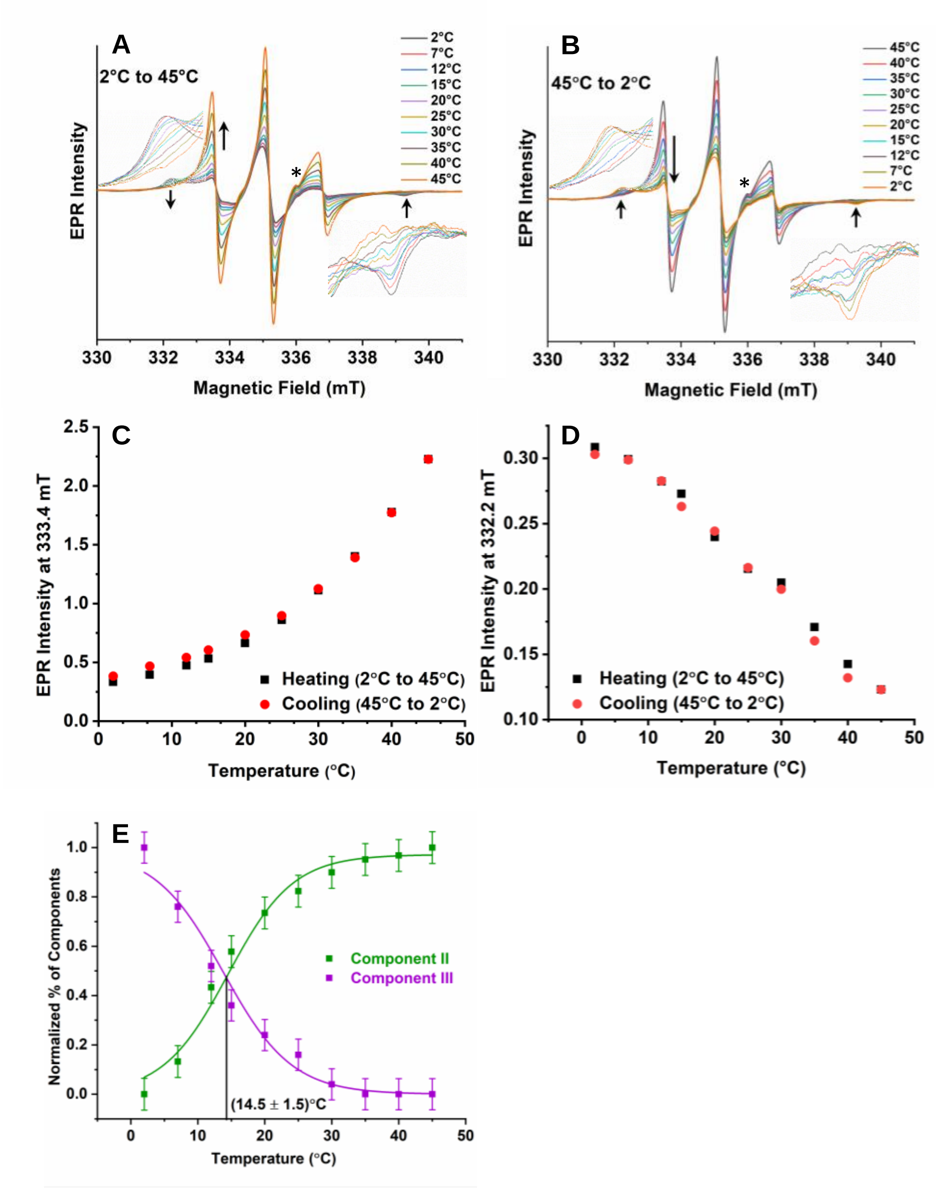

Figure S11. A) EPR spectra of 112 μM CPEB4*,pH8, in 100 mM NaCl upon heating from 2°C (black) to 45°C (orange). B) EPR spectra of the same sample upon cooling from 45°C to 2°C. The insets show a magnification of the regions marked with short arrows. * marks a cavity background signal. C) Plot of the EPR intensity at 332.2 mT and D) 333.4 mT upon heating (black) and cooling (red) obtained from panels A and B. E) Normalized percentages of components **II** and **III** as a function of temperature for 112 μM CPEB4* (green and purple) and a sigmoidal fit.

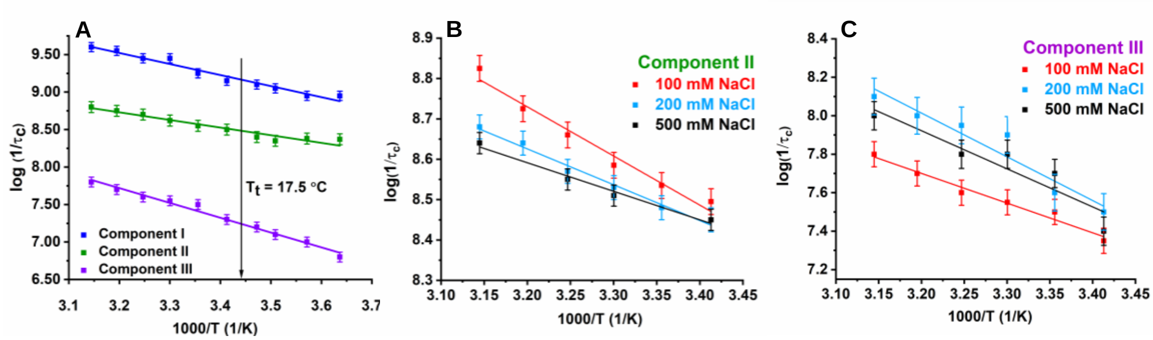

Figure S12. A) Plot of log(1/τ_c) at different temperatures for component **I**, **II** and **III** for 112 µM CPEB4* with 100 mM NaCl. The arrow indicates T_t as determined from Fig. S5. The same plot for the three NaCl concentrations for component **II** in B) and for component **III** in C) at different temperatures in the droplet temperature range (20°C to 45°C). The slope of these lines give rotational diffusion activation energy in KJ/mol. The concentration of CPEB4* for 100 mM NaCl was 112 µM and 90 µM for 200 and 500 mM NaCl.

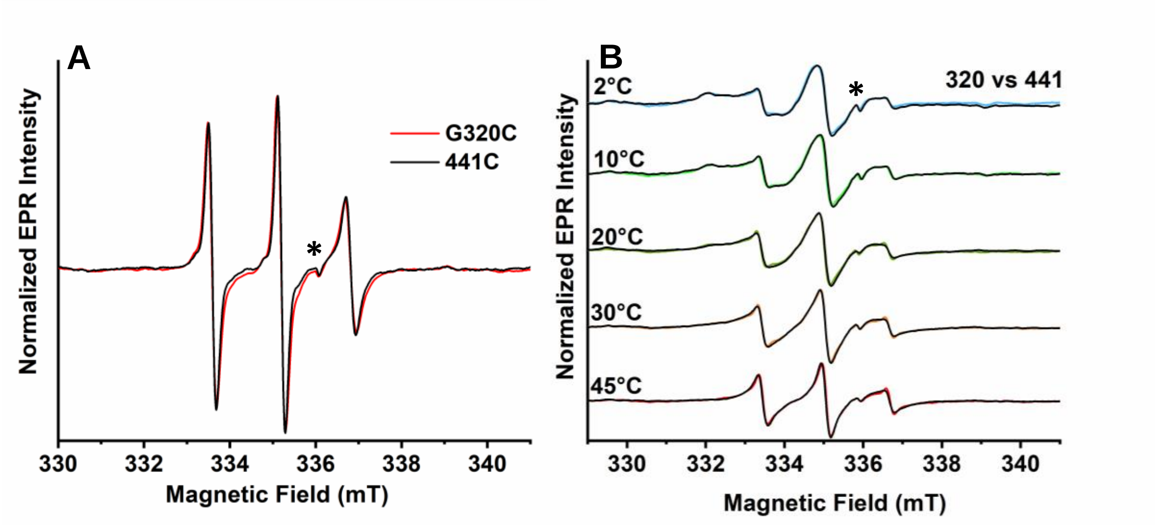

Figure S13. A) Comparison of EPR spectra of MTSL labelled CPEB4$_{NTD}$ at position 441 and 320 and in 3M GdmCl. B) Comparison of the two labeling position in the temperature range of 2- 45°C in 100 mM NaCl. The concentration of the G320C mutant was 40 μM and of 441C is 50 μM. * marks a cavity background signal.

8. EPR Spectroscopy: Effect of concentration

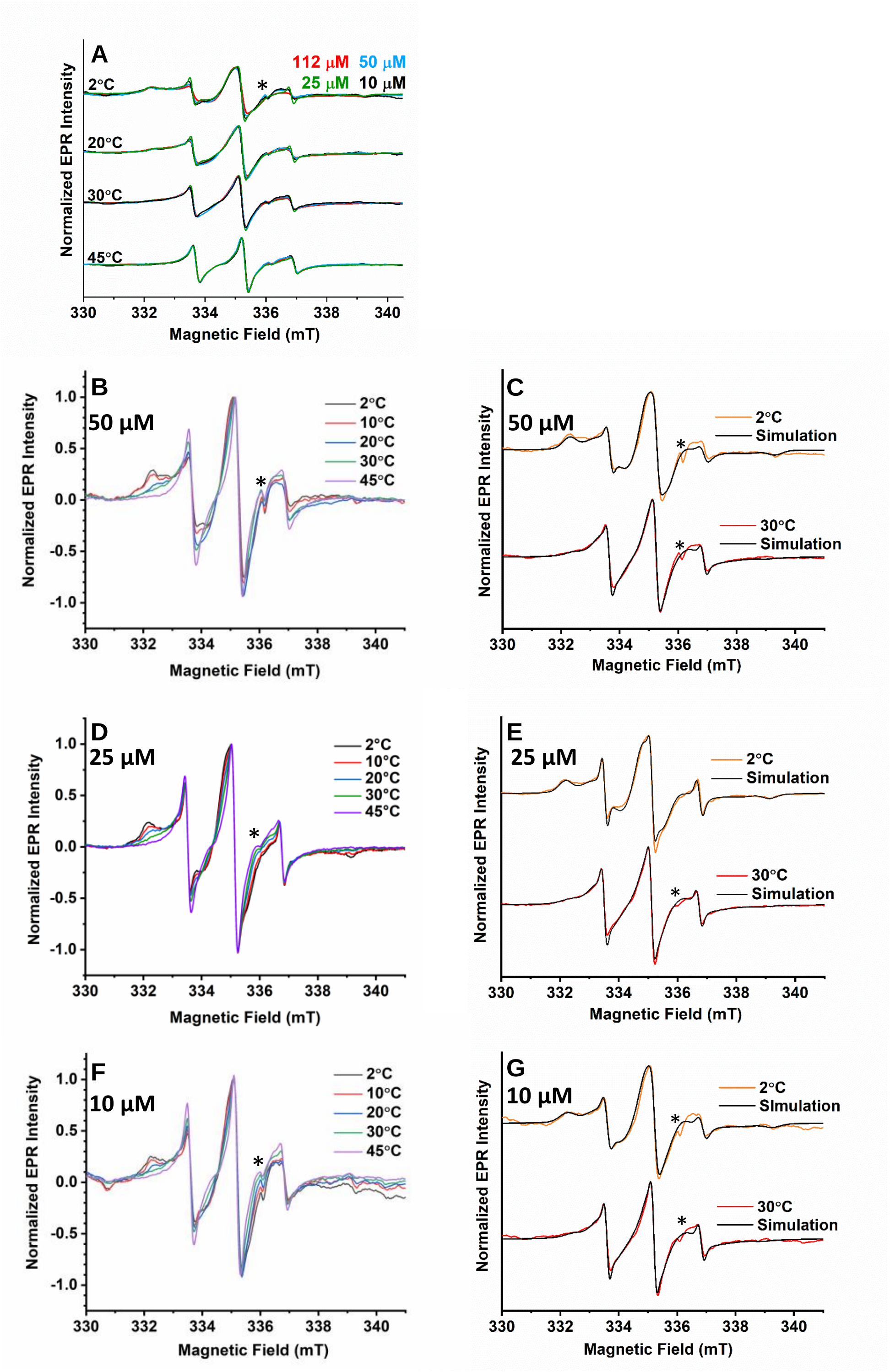

Figure S14. A) EPR spectra of [CPEB4*]= 112 μM (red), 50 μM (cyan), 25 μM (green) and 10 μM (black) at 2°C, 20°C, 30°C and 45°C in 100 mM NaCl. B), D) and F) are EPR spectra of 50 μM, 25 μM and 10 μM CPEB4*, respectively with 100 mM NaCl at different temperatures. Simulation of the EPR spectra of C) 50, E) 25 and G) 10 μM CPEB4* in 100

mM NaCl at 2°C (dark yellow) and 30°C (red). The black spectrum is the corresponding simulation. * marks a cavity background signal.

Table S4: τ_c of component **I** at 2°C for 10, 25, 50 and 112 µM CPEB4* in log scale.

**CPEB4* concentration (µM)**	**10**	**25**	**50**	**112**
log τ_c (τ_c in sec) of component **I** at 2°C	-9.5	-9.45	-9.1	-8.95

9. EPR spectroscopy: Salt effect

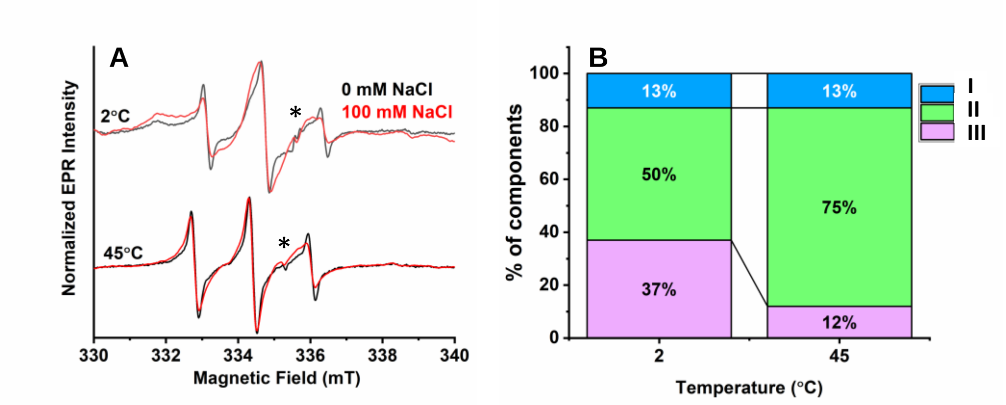

Figure S15. A) EPR spectra of 10 µM CPEB4* in the absence of any salt (black) and in 100 mM NaCl at 2°C (upper) and at 45°C (lower). * marks a cavity background signal. B) Relative amounts (in %) of the three components obtained from the simulations of the 10 µM CPEB4* in 0 mM salt at 2°C and 45°C.

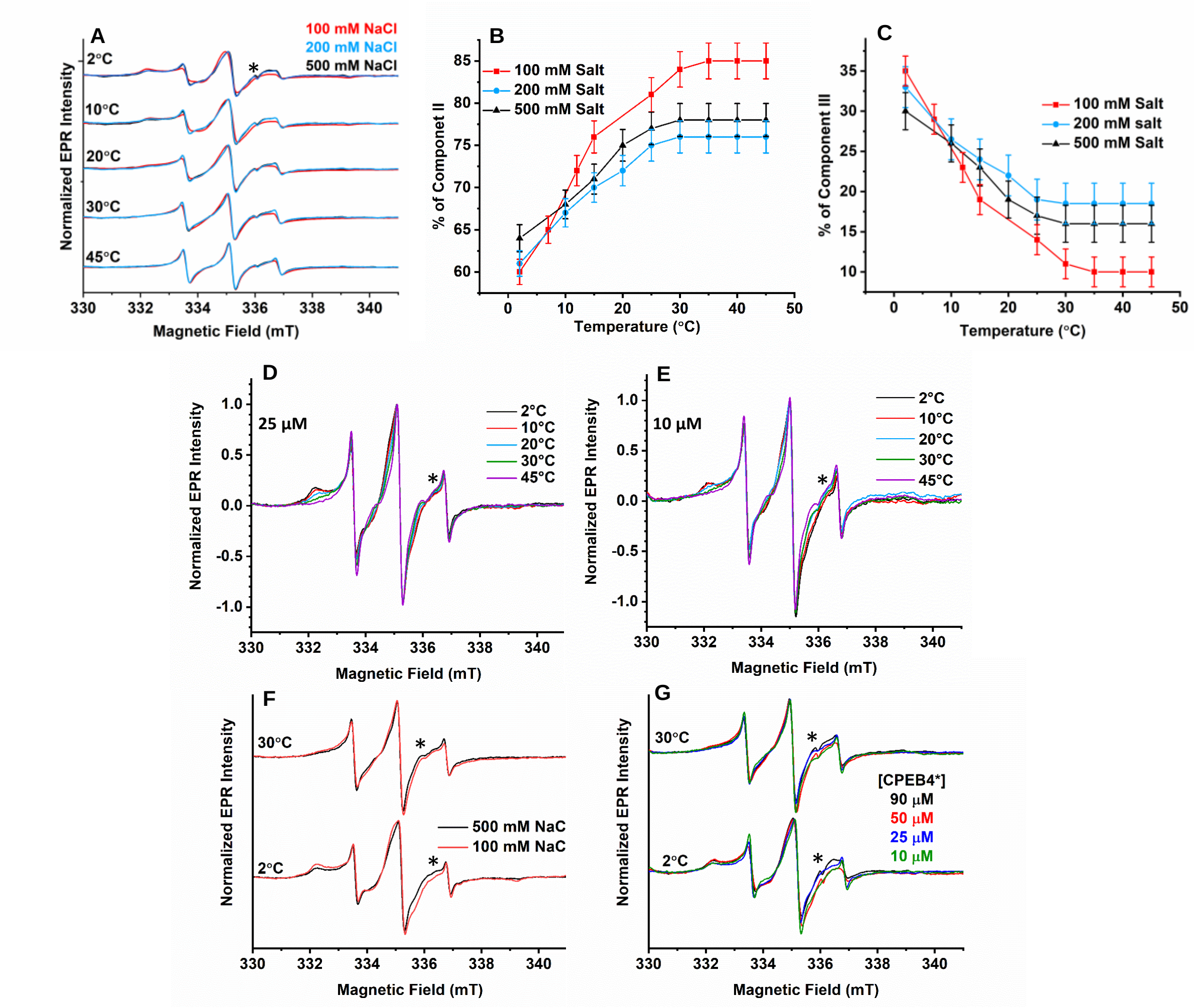

Figure S16. A) EPR spectra of CPEB4* at 100 (red), 200 (cyan) and 500 mM (black) NaCl as a function of temperature. The concentration of CPEB4* for 100 mM NaCl was 112 µM and 90 µM for 200 and 500 mM NaCl . Relative population of B) component **III** and C) components **II** at different NaCl concentrations versus temperature. D) and E) EPR spectra of 25 and 10 µM CPEB4*, respectively at different temperatures in 500 mM NaCl. F) Comparison of the EPR spectra of 25 µM CPEB4* at 2°C (lower) and 30°C (upper) in 100 (red) and 500 (black) mM NaCl. G) Comparison of the EPR spectra at 2°C and 30°C for 90, 50, 25 and 10 µM CPEB4* in 500 mM NaCl. * marks a cavity background signal.

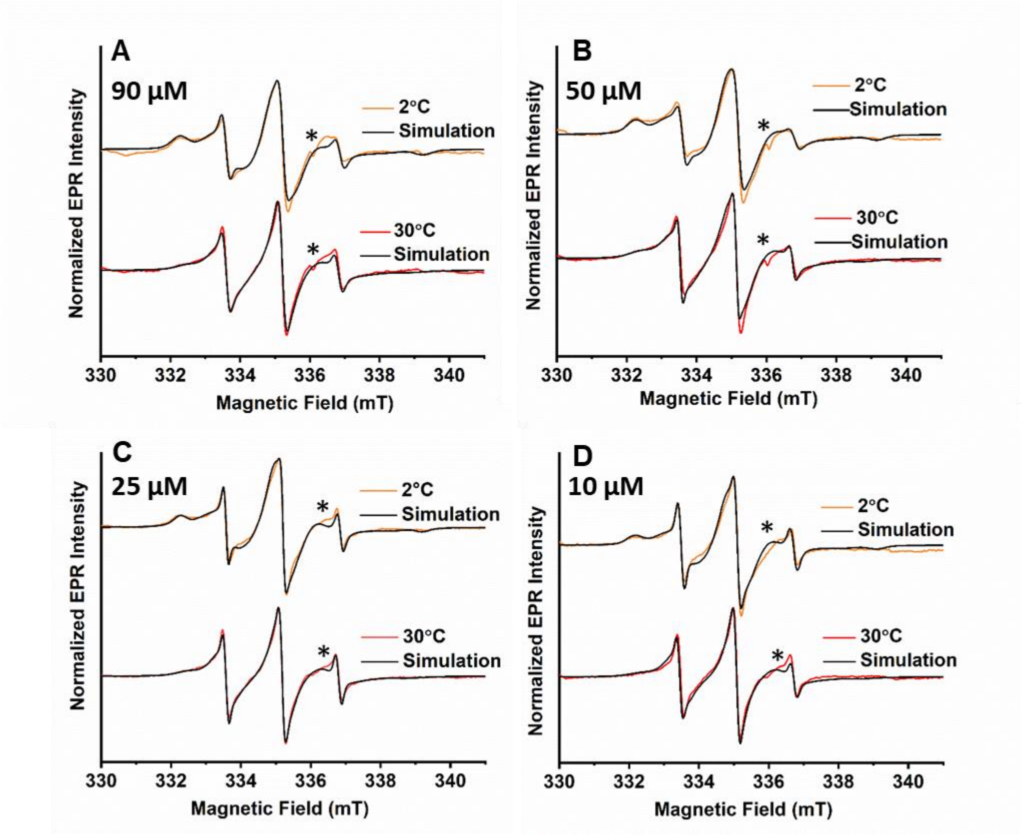

Figure S17. Simulation of the EPR spectra of A) 90, B) 50, C)25 and D) 10 µM CPEB4* in 500 mM NaCl at 2°C (dark yellow) and 30°C (red). The black spectrum is the corresponding simulation. * marks a cavity background signal.

10. Interaction with 1,6 Hexanediol

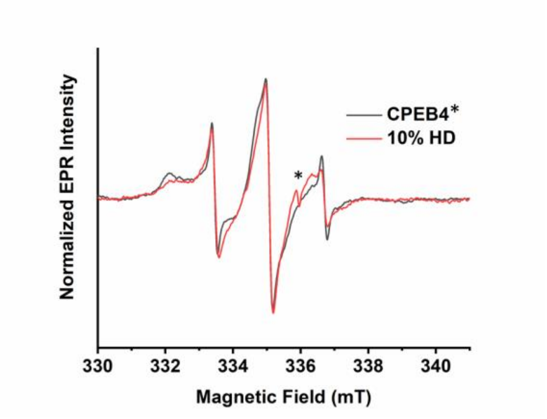

Figure S18. EPR spectra of 20 µM CPEB4* and in the presence of 10% 1,6-hexanediol (HD) (red) at 2°C. * marks a cavity background signal.

Table S5: Amino acid composition of CPEB4$_{NTD}$. The hydrophobic residues are highlighted in red.

Amino acid	Number	Amino acid	Number	Amino acid	Number
Ala	34	His	27	Ser	58
Arg	18	Ile	17	Thr	18
Asn	30	Leu	28	Tyr	6
Asp	17	Lys	14	Val	6
Gln	32	Met	5	Trp	6
Glu	19	Phe	24	Cys	1
Gly	43	Pro	45		
